# Photoswitchable Peptides as Molecular Tools to Encode Structural Order and Disorder in Intracellular Assemblies

**DOI:** 10.1002/anie.202514781

**Published:** 2025-12-04

**Authors:** Julian Link, Luca Burg, Sarah Chagri, Ha‐Chi Nguyen, David Y.W. Ng, Bart Jan Ravoo, Tanja Weil

**Affiliations:** ^1^ Max Planck Institute for Polymer Research Ackermannweg 10 55128 Mainz Germany; ^2^ Center for Soft Nanoscience Universität Münster Busso‐Peus‐Strasse 10 48149 Münster Germany

**Keywords:** Disassembly, Intracellular Self‐Assembly, Light‐responsive systems, Photoswitchable Peptides, Structural Order–disorder

## Abstract

Understanding how self‐assembled structure formation affects cells remains a central challenge in supramolecular chemistry. However, chemical tools that allow access to both ordered and disordered intracellular assemblies from a single molecular scaffold are rare due to design complexity. Here, we present a photoswitchable isotripeptide incorporating an arylazopyrazole (AAP) unit, which undergoes intracellular cleavage to yield a self‐assembling monomer. Upon photoisomerization, the planar *trans*‐isomer forms *β*‐sheet‐rich nanofibers with strong aromatic interactions, while the non‐planar *cis*‐isomer assembles into disordered, random‐coil aggregates lacking aromatic contribution. The structural dynamics of the assemblies are demonstrated by repeated photoswitching between the two states in buffered conditions. Notably, A549 cancer cell viability correlates with the isomer‐dependent assembly behavior and critical aggregation concentrations (CACs): the *trans*‐isomer, with higher aggregation propensity, exhibits greater cytotoxicity. This photoswitchable peptide system thus provides a powerful platform with fast, reversible and robust switching kinetics, long isomer half‐lives, and high photostability to probe the intracellular consequences of supramolecular order and disorder using a single molecular scaffold.

## Graphical abstract

We present a photoswitchable peptide conjugate which exhibits isomerism‐dependent self‐assembly: the planar *trans*‐isomer assembles into well‐ordered nanofibers while the non‐planar *cis*‐isomer yields disordered aggregates. Our study offers a direct visualization on the effects of structure formation inside living cells.

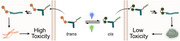

## Introduction

Artificial self‐assembling peptides (SAPs) have emerged as a versatile class of programmable molecules capable of forming morphologically diverse nanostructures with various functions spanning from biological regulation to materials science.^[^ ^1^, ^2^, ^3^ ^]^ Their ability to mimic cytoskeletal elements such as actin filaments^[^ ^4^, ^5^ ^]^ or mediate dynamic interactions akin to intrinsically disordered domains^[^ ^6^, ^7^ ^]^ underscores their importance as bioinspired molecular tools to understand and modulate cellular functions. Synthetic modifications further extend the functionality of SAPs, allowing incorporation of stimuli‐responsive elements, including pH‐sensitive hydrazones,^[^ ^8^, ^9^ ^]^ ROS‐cleavable boronate esters,^[^ ^10^, ^11^, ^12^ ^]^ and photocleavable cages^[^ ^13^, ^14^ ^]^ thereby enabling spatial and temporal control over assembly behavior, structure formation, and cellular interactions.^[^ ^15^ ^]^

Among these systems, *β*‐amyloid‐like motifs have gained prominence due to their well‐defined self‐assembly characteristics and growing utility beyond pathological models.^[^ ^16^, ^17^ ^]^ Applications of SAPs now extend to piezoelectric nanodevices,^[^ ^18^, ^19^ ^]^ nanoscale delivery vehicles,^[^ ^20^, ^21^, ^22^ ^]^ catalysis^23^, ^24^ and responsive biomaterials.^[^ ^25^, ^26^ ^]^ The biological impact of such artificial assemblies is increasingly recognized to depend on their diverse supramolecular architectures and their inherent assembly dynamics as well as other parameters such as intracellular distribution,^[^ ^27^, ^28^ ^]^ biomolecular recognition,^[^ ^29^, ^30^ ^]^ and signal transduction.^[^ ^31^, ^32^ ^]^ However, chemical tools that use a single molecular scaffold to access both ordered and disordered intracellular assembly states remain scarce. Developing such systems is essential for disentangling how structural order governs cellular responses, ultimately guiding the rational design of self‐assembling peptides for functional applications in biology and medicine.

The supramolecular organization of self‐assembling monomers is dictated by a delicate interplay of non‐covalent forces, including hydrogen bonding, van der Waals interactions, hydrophobic interactions, and *π–π‐*stacking.^[^ ^33^, ^34^ ^]^ These interactions are highly sensitive to molecular geometry, such that even minor structural variations can lead to pronounced differences in assembly behavior.^[^ ^35^, ^36^ ^]^ Conventional strategies to modulate this behavior often rely on dynamic covalent chemistry or cleavable functional groups, which, while effective, can increase synthetic complexity and introduce potentially disruptive by‐products.^[^ ^10^, ^11^ ^]^ Non‐assembling control peptides used in these studies typically involve increasing the solubility by substituting hydrophobic amino acids for more hydrophilic ones. Although they function well as controls at the supramolecular level (assembling vs non‐assembling), the molecular structural difference is often not considered or accounted for. In this regard, an emerging alternative exploits molecular isomerization to regulate phase separation and nanostructure formation without altering molecular composition becomes highly attractive. Among various chemical isomerization technologies, light‐triggered isomerization offers a powerful, non‐invasive approach to reversibly control molecular conformation with exceptional spatial and temporal precision.^[^ ^37^, ^38^ ^]^

When photochemistry is applied to modulate the biological activity of analytes by altering pharmacokinetic or pharmacodynamic properties, it is commonly referred to as photopharmacology.^[^ ^39^, ^40^, ^41^ ^]^ The most widely used tools in this field are photocleavable protecting groups, which enable the precise release of biological active molecules upon irradiation. Photoswitches complement this existing portfolio with their reversible switching capability, making them particularly well suited for probing dynamic processes in living systems.^[^ ^42^ ^]^ As such, photoswitches have been employed to control the activity of ion channels,^[^ ^43^ ^]^ enzymes^[^ ^44^, ^45^ ^]^ or the cytoskeleton.^[^ ^46^ ^]^ A more recent approach to study aggregation processes relevant to Alzheimer's, Huntington's and Parkinson's disease involve the incorporation of unnatural amino acids bearing photoswitchable side chains into the backbone of a small tryptophan zipper peptide, enabling precise control over its secondary structure.^[^ ^47^ ^]^

Several photoswitchable scaffolds, such as azobenzenes,^[^ ^48^, ^49^ ^]^ spiropyrans,^[^ ^50^, ^51^ ^]^ diazocines,^[^ ^38^, ^52^ ^]^ and arylazopyrazoles (AAPs)^[^ ^38^, ^53^, ^54^, ^55^ ^]^ have been developed for this purpose. Although spiropyrans undergo substantial polarity and geometry changes upon ring opening, their limited aqueous stability can hinder biological applications.^[^ ^56^, ^57^ ^]^ Diazocines offer enhanced photostability and thermodynamic control but are more challenging to synthesize and often lack efficient bidirectional switching.^[^ ^58^ ^]^ In contrast, AAPs combine straightforward synthesis, efficient two‐way switching, extended thermal half‐lives, and excellent photostability.^[^ ^55^, ^59^, ^60^ ^]^ Their stability in aqueous and reducing conditions was demonstrated in cyclodextrin‐based systems,^[^ ^54^ ^]^ heparin‐conjugates,^[^ ^61^ ^]^ and opioid‐receptor activation^[^ ^62^ ^]^ and makes them especially attractive for biological systems.^[^ ^63^ ^]^ Moreover, the potential of AAPs for photoresponsive supramolecular self‐assembly in aqueous media has been demonstrated,^[^ ^64^, ^65^ ^]^ however, their integration into live‐cell environments for dynamic control of supramolecular behavior has yet to be fully explored.

Herein, we report a photoswitchable isotripeptide incorporating an arylazopyrazole (AAP) unit that undergoes intracellular activation to yield photosensitive monomers with different aggregation dynamics and assembly morphology. This minimalistic construct enables reversible access to distinct supramolecular states, either ordered fibrillar nanostructures or disordered aggregates, through light‐controlled isomerization. The assembly behavior is governed by conformation‐dependent steric effects, while the overall chemical constitution of the peptide remains unchanged between isomers. This design allows for a direct interrogation of how molecular geometry influences self‐assembly behavior and its functional consequences for the interaction with biological structures inside living cells. By further integrating redox‐ and pH‐responsive elements into the peptide‐AAP framework, we establish a modular platform for an environment‐sensitive assembly within complex cellular contexts. This work lays the foundation for spatiotemporally resolved studies of supramolecular organization and its role in cellular processes, advancing the application of dynamic molecular systems in living environments.

## Results and Discussion

### Design of the pH‐ and Glutathione‐Responsive Photoswitchable AAP‐ISA Tripeptide

The modified tripeptide AAP‐ISA is designed as a minimalist model of a peptide photoswitch capable of forming distinct, isomer‐dependent nanostructures driven by differences in steric demand of the aromatic head group. It features the pro‐assembling peptide sequence Ile‐Ser‐Ala, which can self‐assemble into nanofibers when a fluorenylmethoxycarbonyl (Fmoc) *π*‐block is conjugated to its *N*‐terminus.^[^ ^11^ ^]^ Replacement of the Fmoc‐unit with an AAP photoswitch enables light‐induced switching between a *trans*‐isomer (**1*_trans_***) and a *cis*‐form (**1*_cis_***). In the planar *trans*‐form, efficient *π‐π*‐interactions support self‐assembly, whereas the steric kink of the *cis*‐isomer disrupts stacking, thus preventing ordered self‐assembly.

**Figure 1:**
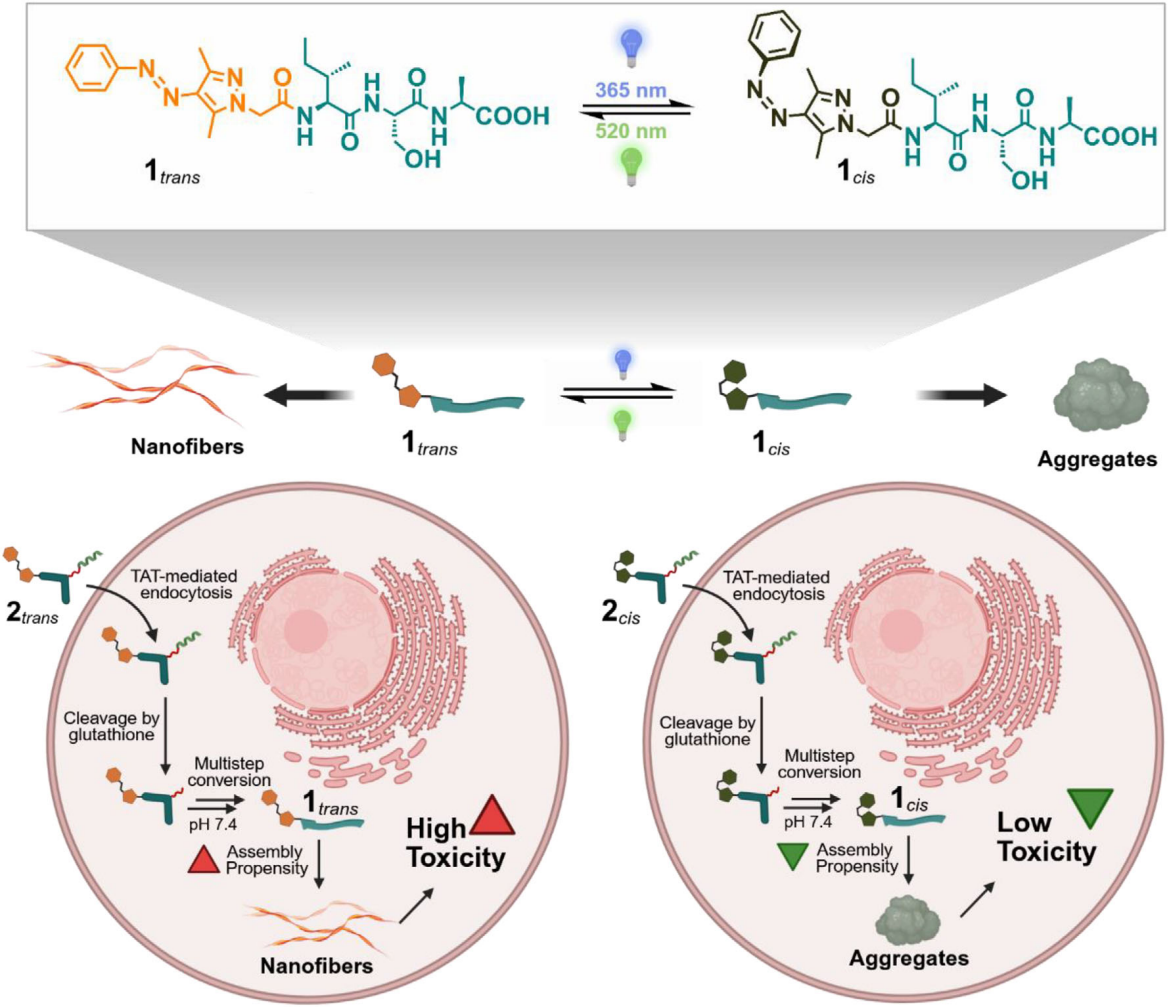
Schematic illustration of the photoswitchable peptide AAP‐ISA (**1**) that self‐assembles into a light‐responsive phase separating material. The resulting nanostructure is dependent on the respective isomer of the photoswitch unit. Extending this peptide toward bioactivity leads to a compound that can enter the cell, be cleaved by glutathione and release **1** in a multistep conversion at physiological pH. The *trans*‐isomer assembles into ordered nanofibers, whereas the *cis*‐isomer forms disordered aggregates. Both isomers induce different cellular responses. Figure created with Biorender.com.

**Figure 2:**
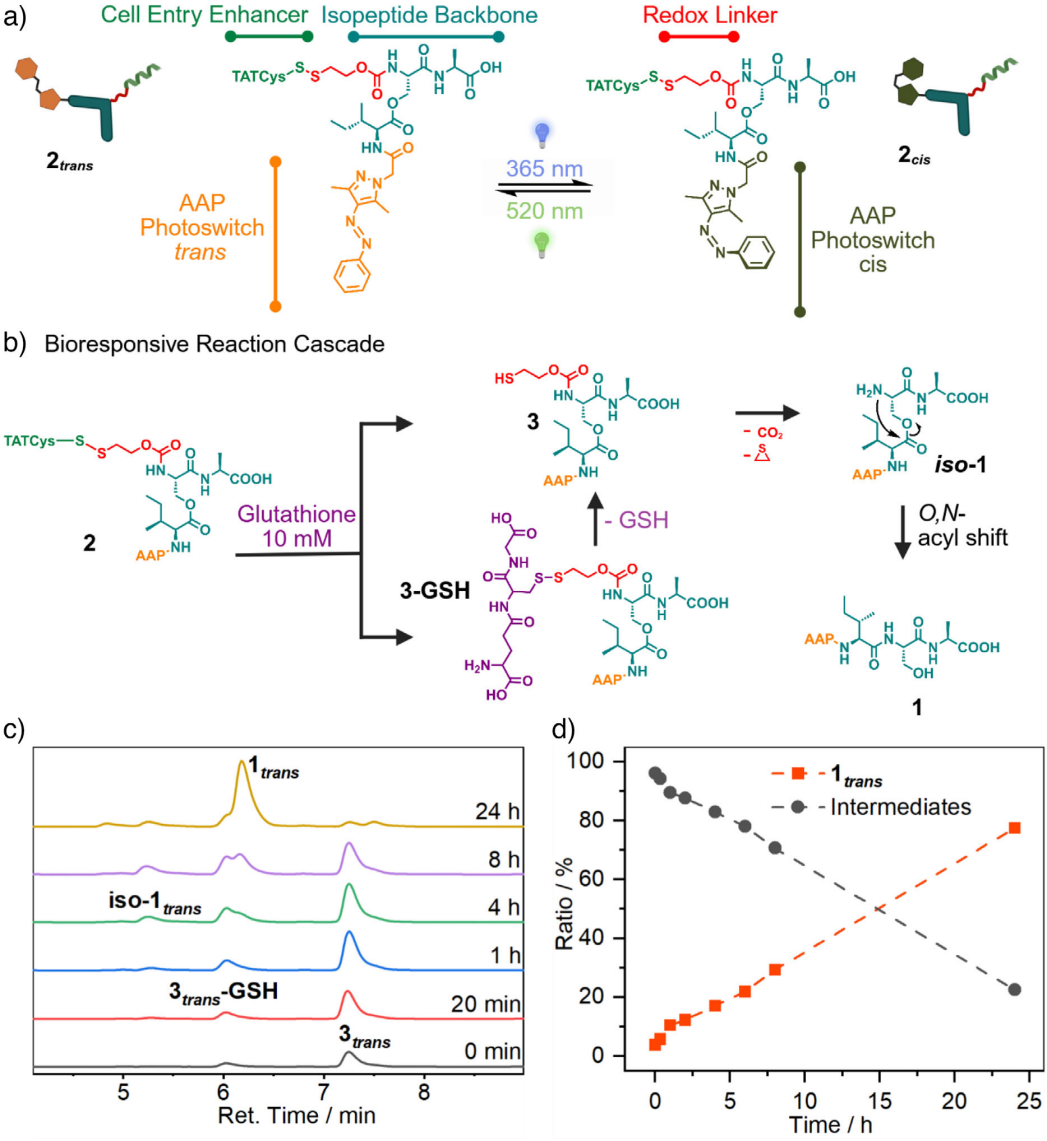
**a**) Chemical design of the triple‐responsive isopeptide (**2**) composed of the cell‐penetrating CysTAT peptide (green) attached to the self‐assembly promoting iso‐ISA peptide backbone (blue) via a redox sensitive disulfide linker (red) and the AAP photoswitch (orange/dark green). Upon irradiation with light at 365 nm the *trans*‐*cis*‐isomerism (**2*_trans_*** → **2*_cis_***) and at 520 nm the *cis*‐*trans*‐isomerism (**2*_cis_*** → **2*_trans_***) can be induced. **b**) Visualization of the chemical transformations within the bioresponsive reaction cascade. First, cleavage of the disulfide linker by glutathione, followed by self‐immolation of the linker. Second, intramolecular rearrangement by *O,N*‐acyl shift yielding the linearized AAP‐ISA (**1**). **c**) LC‐MS kinetic analysis of the glutathione‐induced linearization of native **2*_trans_*** in NH_4_HCO_3_ buffer (50 mM, pH 7.4) and methanol (v/v 1:1) in the presence of intracellular concentrations of glutathione (10 mM) at room temperature. Corresponding masses can be found in the Supporting Information (Figure S31) **d**) Relative ratio of intermediates (**iso‐1**, **3**, **3‐GSH**) and final product **1** after the addition of glutathione‐containing buffer, based on the peak integration at 300 nm. The data shown here is derived from the native *trans*‐isomer. The corresponding data for the *cis*‐isomer can be found in the supporting information (Figure S32).

To enable cellular uptake, the linear peptide sequence has to be modified toward cell‐penetrating capability. First, the structure of the peptide core is altered into a pro‐assembling kinked isopeptide (Figure 2a, blue). A glutathione‐responsive self‐immolative linker is then attached to the *N*‐terminus (Figure 2a, red). This linker is conjugated via a disulfide bond to a cysteine‐extended cell‐penetrating peptide (Figure 2a, green), specifically the trans‐activator of transcription (TAT). Upon TAT‐mediated uptake, the disulfide linker is cleaved by intracellular glutathione (GSH), yielding the kinked intermediate **3** (Figure 2b). Self‐immolation of the linker is then triggered, resulting in the release of the isopeptide **iso‐1**, as also demonstrated in previous studies.^[^ ^15^, ^66^ ^]^ Lastly, the kinked peptide backbone undergoes an *O,N*‐acyl shift, yielding the linearized monomer **1** (Figure 2b). This monomer subsequently self‐assembles into either nanofibers (*trans‐*isomer) or amorphous aggregates (*cis*‐isomer, Figure 1). The kinetics of this transformation, both *cis*‐ and *trans*‐isomer, in the presence of intracellular concentrations of GSH (10 mM)^[^ ^67^ ^]^ were monitored by liquid chromatography coupled with mass spectrometry (LC‐MS) in a 1:1 mixture of NH_4_HCO_3_ buffer (50 mM, pH 7.4.) and methanol, ensuring the solubility of all components (Figure 2c). Immediate cleavage of the disulfide bond occurred followed by the formation of the isopeptide bearing the disulfide linker (**3**, *t_R_* = 7.5 min) and the corresponding GSH‐conjugate (**3‐GSH**, *t_R_* = 6.3 min). Over 24 h, the peptide gradually converted into the free isopeptide (**iso‐1**, *t_R_* = 5.5 min) and subsequently into the linearized monomer **1** (t_R_ = 6.5 min) with both isomers achieving similar kinetic profiles (Figure 2d and Figures S31 + 32).

### Molecular Characteristics and Photophysical Properties of the Photoswitchable Tripeptide 1

**Figure 3:**
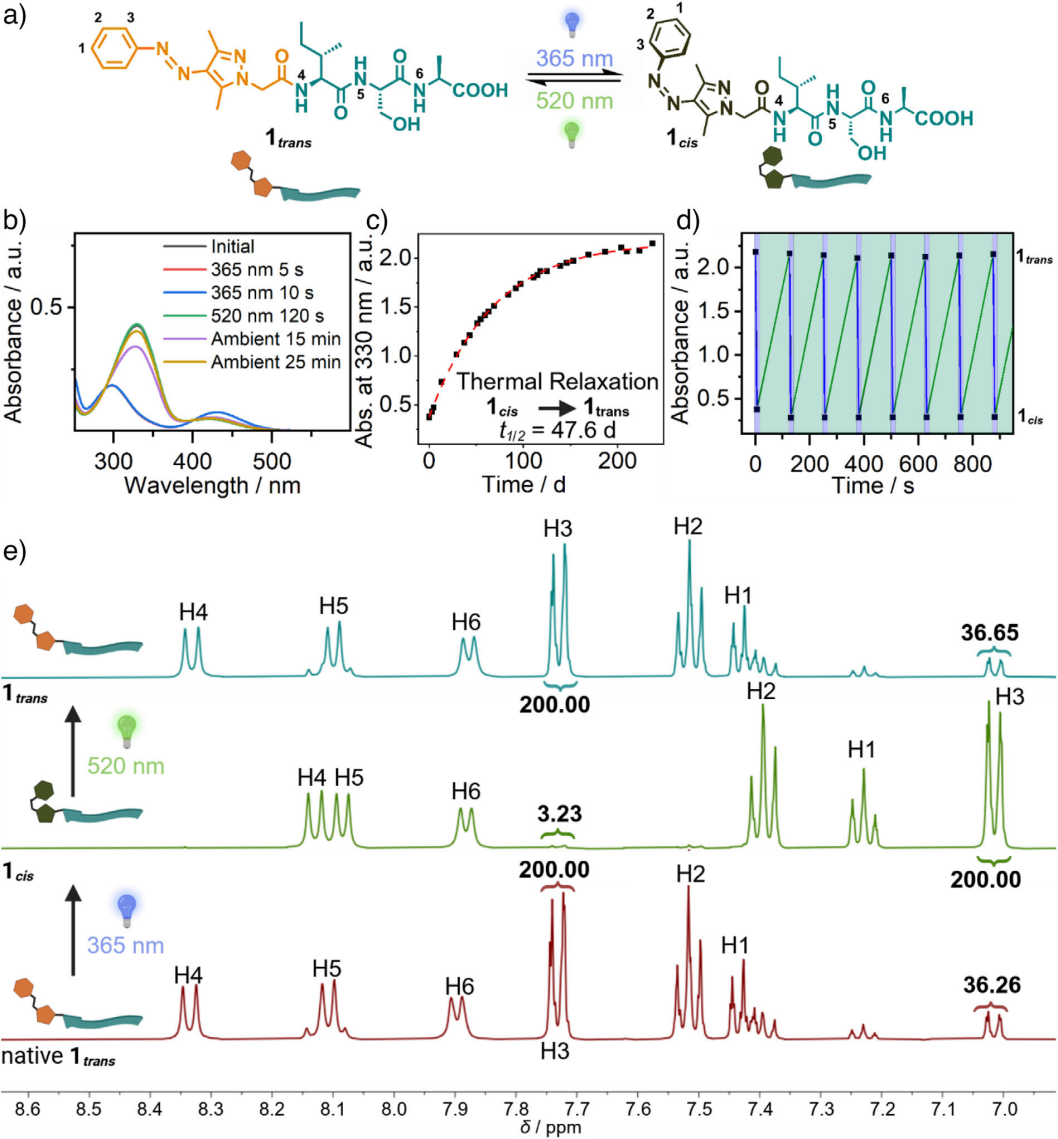
**a**) Chemical structures of AAP‐ISA in *trans*‐ (**1*_trans_***) and *cis*‐state (**1*_cis_***). **b**) UV–vis absorbance spectra of AAP‐ISA (**1**) over different irradiation times at different wavelengths. **c**) Half‐life time in the dark at ambient conditions for **1*_cis_***. **d**) UV–vis absorbance of **1** over the course of 10 irradiation cycles at 365 nm for 5 s (indicated by blue box) and 520 nm for 120 s (green box). **e**) ^1^H‐NMR spectroscopy (DMSO‐d_6_, 298K, 400 MHz, 2 mg ml^−1^) analysis of AAP‐ISA in native state (**1*_trans_***, red), after irradiation at 365 nm for 15 s (**1*_cis_***, green) and after irradiation at 520 nm for 240 s (**1*_trans_***, blue). Integrals were set to 200.00 for the respective H3‐protons of the predominant isomer.

The key characteristic of a photoswitch is its ability to reversibly transform between isomers upon irradiation. Previous studies on the AAP demonstrated that irradiation with UV light at 365 nm converts the native *trans*‐isomer to the *cis*‐isomer, while green light (520 nm) induces the reverse isomerization.^[^ ^53^ ^]^ This reversible *trans*‐to‐*cis* transition is marked by a decrease and a hypsochromic shift of the π→π* band from 330 to 298 nm, along with an increase and bathochromic shift of the n→π* band from 413 to 431 nm. UV–vis spectroscopy (Figure 3b) revealed that the *trans*→*cis* isomerization was complete within 5 s, whereas the *cis*→*trans* transformation required approximately 120 s (Figure 3b). The thermal relaxation half‐life in the dark at ambient conditions was determined to be 47.6 days, providing a stable time window for all subsequent experiments (Figure 3c). Remarkably, over the course of 10 switching cycles, the fidelity of **1*_trans_*** is almost quantitatively preserved (>98%) (Figure 3d).

Switching ratios of **1** were quantified with ^1^H‐NMR‐spectroscopy in DMSO‐d_6_, in which no self‐assembly occurs (Figure 3e). The irradiation durations used in the NMR studies (15 s (365 nm), 240 s (520 nm)) were increased due to the higher concentrations (∼3.8 mM) required for obtaining sufficient resolution compared to the other experiments (5 s (365 nm), 120 s (520 nm), 100–400 µM). The spectra were recorded immediately after the respective irradiation time intervals (Figure 3e, red→green). Even after 30 min of irradiation (520 nm), the ratios of **1*_trans_*** versus **1*_cis_*** did not change further (respective ratio of H3 protons 200.00:36.65), indicating completed conversion of **1*_cis_*** to **1*_trans_*** already after 240 s (Figure S30). Comparing the aromatic phenyl protons of **1**, the signals of the *trans*‐state at 7.73, 7.51, and 7.43 ppm nearly vanished, while three new signals at lower chemical shift values at 7.01, 7.39, and 7.23 ppm emerged corresponding to the respective phenyl protons of the *cis*‐isomer. Additionally, the isoleucine and serine amide peaks shifted from 8.33 and 8.10 ppm to 8.13 and 8.08 ppm, respectively. Upon irradiation with green light (520 nm), the initial signals could be recovered, whereas the corresponding *cis*‐signals disappeared (Figure 3e, green→blue). Based on peak integration in the native‐state (Figure 3e), 85% of the molecules were in the *trans*‐state (**1*_trans_***) and 15% were in the *cis*‐configuration (**1*_cis_***). After irradiation with light at 365 nm, >98% of **1*_trans_*** transitioned to the *cis*‐state (**1*_cis_***), with only <2% remaining in *trans*‐form (**1*_trans_***). Subsequent irradiation with green light restored the native *trans*‐state (87% *trans*‐state, 13% *cis*‐form). These results for absorbance, chemical shifts and switching efficiencies are in alignment with the data set reported for the free AAP carboxylic acid.^[^ ^53^ ^]^

### Programming Supramolecular Order Versus Disorder of the Photoswitchable Tripeptide 1

The peptide assemblies were analyzed using circular dichroism (CD) spectroscopy, transmission electron microscopy (TEM), and a dynamic light scattering (DLS) aggregation assay as well as Proteostat aggregation assay. In our initial investigations, we examined the self‐assembly behavior of the molecule in its *trans* and *cis* isomeric states independently, with the photoisomerization occurring prior to the assembly process. This approach allowed us to examine how each molecular state influences the structural organization and intermolecular interactions during self‐assembly.

We prepared DMSO stock solutions of both isomers respectively, and diluted them to the effective conditions of self‐assembly (99% buffer, 1% DMSO). TEM imaging in phosphate buffered saline (PBS) revealed that the *trans*‐isomer formed twisted, tubular‐like networks at concentrations ranging from 150 to 400 µM (Figure S22). These structures appear to arise from backbone alignment facilitated by *π‐π*‐interactions of the planar, hydrophobic *trans*‐AAP photoswitch. Following the irradiation at 365 nm for 5 s, these well‐defined architectures were entirely absent, suggesting that the twisted and more polar *cis*‐AAP photoswitch hinders the self‐assembly of the peptide. This behavior remained consistent across various buffer conditions, including hydroxyethylpiperazine ethane sulfonic acid (HEPES) and phosphate buffers supplemented with either chloride or fluoride salts, and in the absence of additional electrolytes (Figure S22).

**Figure 4:**
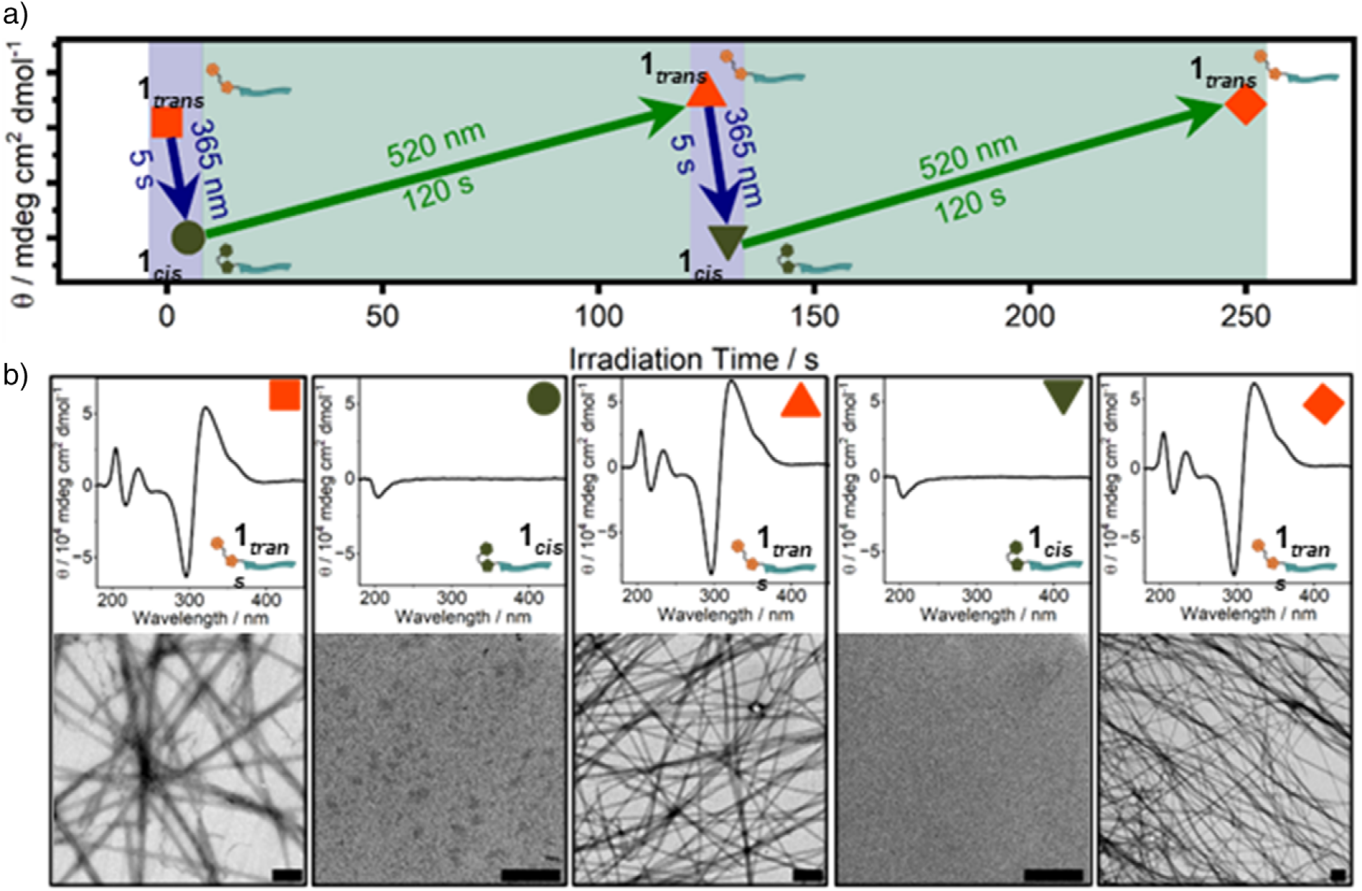
**a**) Molar ellipticity of **1** at 325 nm over the course of 2 irradiation cycles at 365 nm for 5 s (blue box) and 520 nm for 120 s (green box). In between the individual irradiation steps, corresponding TEM micrographs and CD‐spectra at 200 µM in PBS were recorded **b**). Individual measurements are indicated by different symbol shape. Scale bar for TEM‐micrographs = 500 nm.

Building upon these insights, we shifted our focus to dynamic systems where photoisomerization is induced after the assembly has taken place. This enables us to explore how structural transitions propagate through pre‐formed assemblies, offering an understanding of the molecular responsiveness and potential reversibility within these supramolecular architectures. For this, the linear peptide in the native *trans*‐form (**1*_trans_***) was dissolved in PBS at 200 µM and initial TEM images and CD spectra were recorded. At this concentration, the formation of fibrillar structures was observed for **1*_trans_***, while **1*_cis_*** did not show structure formation. The sample was then irradiated for 5 s at 365 nm to initiate *trans*‐*cis*‐isomerism (**1*_trans_*** → **1*_cis_***), followed by back‐irradiation at 520 nm for 120 s to initiate the reverse *cis*‐*trans*‐isomerism (**1*_cis_*** → **1*_trans_***). After the first cycle, a second 365 nm/520 nm irradiation cycle was performed to investigate the reversible behavior and any inherent structural adaptations of the supramolecular assemblies. Between each individual irradiation step, CD‐spectroscopy was measured, and TEM samples were prepared (Figure 4) to analyze if the assembled structures of the *trans*‐isomer could fully disassemble after the photoisomerization into the *cis*‐monomer and reassemble upon the reverse switching. The experimental setup, including the irradiation conditions, is illustrated in Figure 4a. Data points are represented as colored symbols (orange = **1*_trans_***, green = **1*_cis_***) indicating the respective molar ellipticity at 325 nm as a function of total irradiation time.

The native *trans*‐state before any irradiation (Figure 4, orange square) showed dense fibrillar network observed in TEM (Figure 4b), comparable to previously observed results (Figure S22). It was accompanied by CD‐spectroscopy data indicating chiral secondary structure formation due to aromatic interactions and beta‐sheet formation. The fiber‐forming *trans*‐isomer (**1*_trans_***) exhibited a maximum at 204 nm, followed by a minimum at 217 nm, indicating n‐π* transitions associated with the presence of antiparallel *β*‐sheets.^[^ ^68^, ^69^, ^70^, ^71^, ^72^ ^]^ A subsequent positive cotton effect observed at 233 nm (Figure 4, orange square) could be due to *π‐π* stacking of the aromatic AAP photoswitch, a phenomenon also reported in peptides containing aromatic side chains.^[^ ^73^, ^74^ ^]^ Additionally, the CD spectrum highlights a strong aromatic contribution from the photoswitch in the far‐UV region (Figure 4, orange square). A bisignate dichroic peak with a minimum at 296 nm, followed by a maximum at 322 nm and a shoulder at ∼360 nm, likely arises from π‐π* transitions of ordered AAP motifs. This negative‐to‐positive pattern indicates a positive exciton couplet and therefore a right‐handed structure formation.^[^ ^71^ ^]^

UV‐irradiation of **1*_trans_*** converted the *trans*‐ into the *cis*‐isomer (Figure 4, green circle). In TEM, **1*_cis_*** did not assemble into nanostructures (Figure 4, green circle) and the corresponding CD spectrum showed no signals indicating *β*‐sheet structures or aromatic interactions (Figure 4, green circle). Instead, the *cis*‐isomer showed a small CD minimum at 204 nm, suggesting a random coil conformation (Figure 4, green circle).^[^ ^64^ ^]^

However, the reformation of **1*_trans_*** by subsequent irradiation with green light restored these dense fibrillar networks with prominent *β*‐sheet signatures, indicated by a maximum at 204 nm followed by a minimum at 217 nm, as well as aromatic contributions (minimum at 296 nm, maximum at 322 nm) completely (Figure 4, orange triangle). This process proved to be remarkably fast and fully reversible, with complete structural recovery observed after just 5 s (**1*_trans_*** → **1*_cis_***) or 120 s (**1*_cis_*** → **1*_trans_***) of irradiation. The system maintained its responsiveness and structural integrity over 10 cycles of irradiation (Figure S24), demonstrating its robust and dynamic supramolecular character. These observations are consistent with the photoresponsive self‐assembly of AAP‐containing hydrogelators described in previous reports.^[^ ^65^, ^75^ ^]^

Next, DLS and Proteostat assays were employed to determine the critical aggregation concentration (CAC) of the two isomers. For DLS, the mean count rate was measured at different concentrations and the CAC was identified by a steep increase in the count rate, as reported earlier.^[^ ^26^ ^]^ For Proteostat assay, the steep increase of absorbance of the dye indicated the CAC.^[^ ^69^ ^]^ For the *trans*‐isomer, the CAC was found to be ∼160 µM for both methods, while for the *cis*‐isomer, it increased approximately two‐fold, reaching ∼360 µM (Figure S26). To assess whether CD and TEM data reveal significant differences at concentrations above the CAC of **1*_cis_***, the irradiation experiment shown in Figure 4 was repeated at 400 µM (Figure S25). The CD spectra showed no notable changes compared to those obtained at 200 µM. In TEM, the nanostructures formed by **1*_trans_*** were conserved while **1*_cis_*** exhibited the formation of amorphous aggregates. These observations are consistent with the CAC of **1*_cis_*** (Figure S26) and highlight the impact of the photoswitch, regardless of whether the supramolecular assemblies form highly ordered structures (**1*_trans_***) or disordered aggregates (**1*_cis_***).

After analyzing the structure formation of the linear isotripeptide **1** alone in detail, the structure formation upon GSH induced transformation from **2** into **1** was investigated. For this, **2** was incubated in PBS (200 µM) for 24 h and prepared for TEM analysis with and without irradiation for 5 s at 365 nm. The TAT‐modified peptides (**2*_trans_*** and **2*_cis_***) did not exhibit any defined nanostructure formation, regardless of photoisomerization (Figure S23I and J). However, after incubation with GSH (10 mM, 0–24h), the *trans*‐isomer gradually formed nanofibers, whereas the *cis*‐isomer did not (Figure S23), indicating successful release of **1*_trans_*** and **1*_cis_***.

### Photoisomerism Determines Cellular Toxicity of the Isotripeptide 2

To enable fluorescence visualization of the assembly, co‐assembly with Cyanine 5 (Cy5) modified Fmoc‐ISA was performed (**Cy5‐peptide**, Figure S27). Prior to cell experiments, we investigated the successful co‐assembly in a 99/1 mixture of linear **1** and **Cy5‐peptide** using both TEM and confocal microscopy at 200 µM in PBS (pH = 7.4) with 1% DMSO (Figure S28 + 29). TEM images revealed that the formed structures were visually indistinguishable from those of the pure AAP‐ISA system, while confocal microscopy and fluorescence phase contrast microscopy showed fluorescent fibrous structures, supporting a successful co‐assembly of the assembling monomer with the dye‐containing **Cy5‐peptide** (Figure S28 + 29).

Next, we investigated the cellular uptake of the TAT‐bearing bioactive peptides and their subsequent GSH‐induced transformation into the self‐assembling linear peptide inside human lung adenocarcinoma cells (A549). Cells were treated with 100, 200, and 400 µM of the two different isomers of the TAT‐bearing peptides in a 99/1 ratio (**2**/ **TAT‐Cy5‐peptide**), respectively, incubated for 4 h, and analyzed using confocal laser scanning microscopy (CLSM). To better visualize cell morphology, cells were stained with MitoTracker Orange and NucBlue.

**Figure 5:**
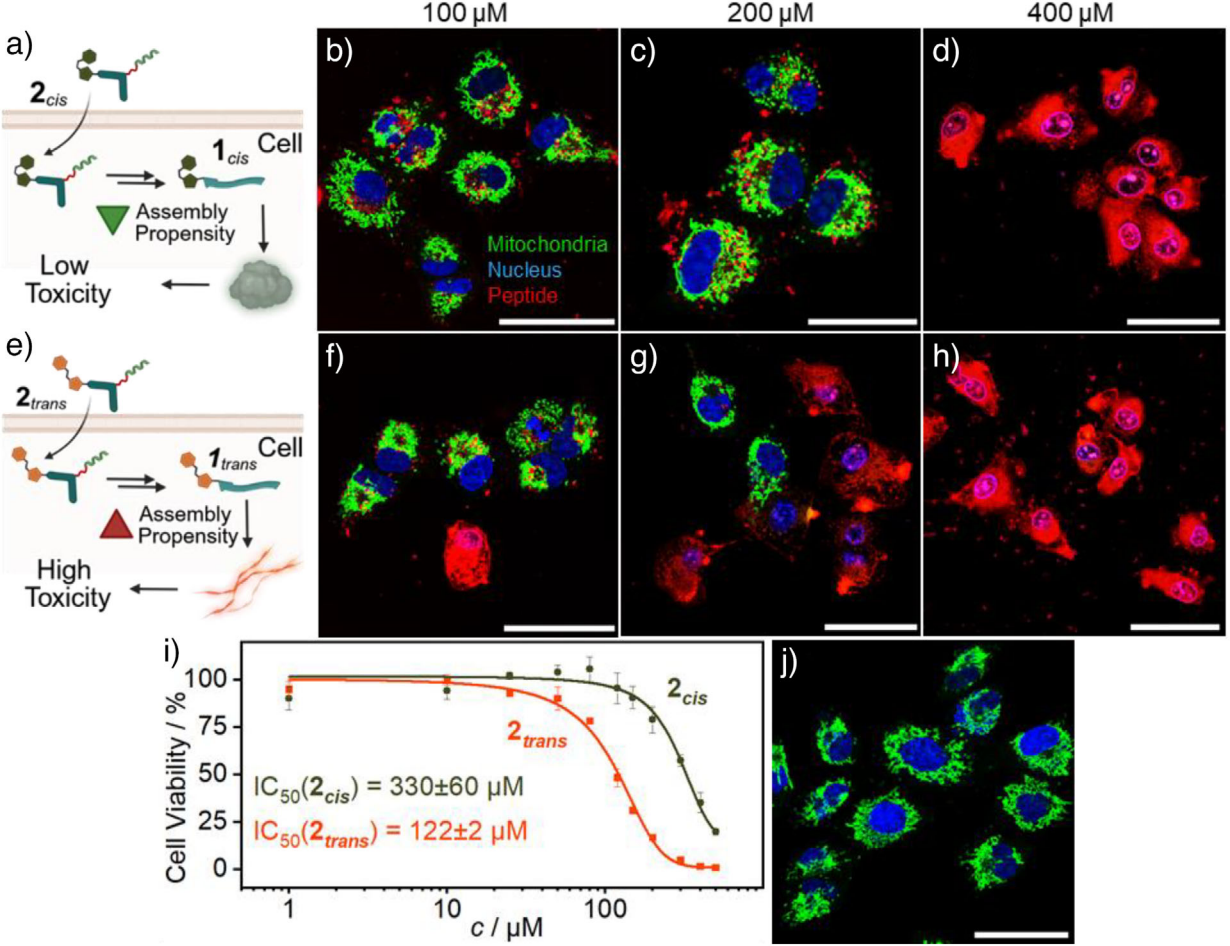
Schematic illustration of the internalization and assembly into nanostructures **a**) **2*_cis_*** → **1*_cis_***, **e**) **2*_trans_*** → **1*_trans_***. Confocal laser scanning micrographs of A549 cells treated for 4h with **2*_cis_*** (100 **b**), 200 **c**) or 400 µM **d**) or with **2*_trans_*** (100 **f**), 200 **g**) or 400 µM **h**)) or without any compound **j**) and prestained with Mitotracker Orange and NucBlue. For visualization, 1% of **TAT‐Cy5‐peptide** (compare Figure S27) was added and coincubated with **2**. Imaging under physiological condition (5% CO2, 90% humidity, and 37°C) using Leica STELLARIS 8 microscope, HC PL APO CS2 40x/1.25 GLYC. Imaging: 550 nm at 2.13% for MitoTracker Orange excitation, 405 nm at 2.23% for NucBlue excitation and 650 nm at 0.66% for sample excitation. Scale Bars: 50 µm. **i**) Cell viability of A549 cells incubated with **2*_cis_*** (green curve) or **2*_trans_* **(orange curve) for 4 h. The IC_50_ value of **2*_trans_*** is 122 ± 2 µM and of **2*_cis_*** is 330 ± 60 µM. Data are presented as mean ± s.e.m, n=4.

Confocal imaging confirmed cellular uptake for both isomers at various concentrations, as evidenced by strong Cy5 fluorescence (Figure 5d–l and Figure S33). No specific subcellular localization was detected, consistent with the ubiquitous cytosolic distribution of GSH.^[^ ^76^ ^]^ The cells treated with 400 µM of both **2*_trans_*** (Figure 5h) and **2*_cis_*** (Figure 5d) showed a strong Cy5 fluorescence indicating a high uptake of the isopeptides. Cells treated at these concentrations showed cell rounding and disruption of mitochondrial network, indicating mitochondrial stress and cell death. For incubation of cells at 100 µM, no dead cells were observed following treatment with the *cis*‐isomer (Figure 5b), indicating no cytotoxicity at this concentration. In contrast, treatment with the *trans*‐isomer at the same concentration resulted in weak toxic effects, like cell rounding and disruption of mitochondrial network (Figure 5g). At 200 µM, the difference between cells treated with the *trans*‐isomer (Figure 5g) versus *cis*‐isomer (Figure 5c) became even more evident: Although cells treated with the *cis*‐isomer remained largely viable, extensive cell death was observed for cells treated with the *trans*‐isomer. These results highlight a marked difference in the cytotoxic profiles of the *cis*‐ and *trans*‐isomers, with the *trans*‐form exhibiting greater potency at lower concentrations.

To quantify these effects, we performed a CellTiter Glo Cell Viability Assay. A549 cells were treated with varying concentrations of both isomers, and the cell viability was assessed via an ATP dependent luminescence measurement after 4 h of incubation (Figure 5c). The *trans*‐isomer exhibited an IC_50_ of 122 ± 2 µM, whereas the *cis*‐isomer showed an IC_50_ of 330 ± 60 µM, representing a 2.7‐fold increase in toxicity. These observations correlate with the differing assembly propensity of the *cis*‐ and *trans*‐peptide isomer and their respective CACs (*trans*‐isomer ∼160 µM, *cis*‐isomer ∼360 µM, Figure S26), suggesting that higher aggregation propensity corresponds to higher cytotoxicity.

These results highlight that even subtle changes in molecular orientation can dramatically alter biological responses due to differences in assembly behavior between the isomers. Here, we designed a peptide system with an identical chemical composition, where the isomer less prone to self‐assemble exhibits lower toxicity compared to the self‐assembling isomer, allowing for the investigation of structure–bioactivity relationships based on no molecular compositional difference.

## Conclusion

In this study, we developed a photosensitive self‐assembling peptide capable of dynamically switching between a disordered and ordered assembled state in response to light. The peptide system displays exceptional photostability and half‐life times, demands only short irradiation times, while offering precise spatiotemporal control over the self‐assembly. Light‐induced switching between distinct *β*‐sheet‐rich nanofiber‐forming structures and disordered random coil states was achieved rapidly and reversibly. Using isopeptide and redox‐sensitive linker chemistry to control intracellular release in cells, this structural transition was further leveraged to modulate bioactivity, demonstrating that the structure‐forming monomer exhibits significantly higher cytotoxicity than its aggregate‐forming counterpart due to its greater propensity for assembly. These findings underscore how differences in superstructural order, without a change in molecular composition, can be controlled by photoisomerization and that cellular behavior toward synthetic structures formed in situ is largely affected by their respective assembly propensities.

## Supporting Information

The authors have cited additional references within the Supporting Information.^[^ ^15^, ^53^ ^]^

## Conflict of Interests

The authors declare no conflict of interest.

## Supporting information



Supporting Information

## Data Availability

The data that support the findings of this study are available in the Supporting Information of this article.
