# Supplementary material for: Photoswitchable Peptides as Molecular Tools to Encode Structural Order and Disorder in Intracellular Assemblies
Source: Angew Chem Int Ed Engl. 2025 Dec 4;65(3):e14781. doi: 10.1002/anie.202514781 (PMC12811659; doi:10.1002/anie.202514781)
Supplement: Supplementary file 1 — Supporting Information [file ANIE-65-e14781-s001.docx]

Supporting Information

Julian Link^[a]^, Luca Burg^[b]^, Sarah Chagri^[a]^, Ha-Chi Nguyen^[a]^, David Y.W. Ng^*[a]^, Bart Jan Ravoo^*[b]^, Tanja Weil^*[a]^

[a] J. Link, Dr. S. Chagri, H.-C. Nguyen, Dr. D.Y.W. Ng, Prof. Dr. T. Weil
Max Planck Institute for Polymer Research
Ackermannweg 10, 55128 Mainz, Germany
weil@mpip-mainz.mpg.de, ng@mpip-mainz.mpg.de

[b] L. Burg, Prof. Dr. B.J. Ravoo
Center for Soft Nanoscience
Universität Münster
Busso-Peus-Strasse 10, 48149 Münster, Germany
b.j.ravoo@uni-muenster.de

Contents

[1. General Information 3](#_Toc201655340)

[1.1 Materials 3](#_Toc201655341)

[1.2 Instruments 3](#_Toc201655342)

[1.2.1 Nuclear Magnetic Resonance (NMR) Spectroscopy 3](#_Toc201655343)

[1.2.2 Liquid Chromatography-Mass Spectrometry (LCMS) 3](#_Toc201655344)

[1.2.3 Transmission Electron Microscopy (TEM) 3](#_Toc201655345)

[1.2.4 Microwave Peptide Synthesizer 3](#_Toc201655346)

[1.2.5 High-Performance Liquid Chromatography (HPLC) 4](#_Toc201655347)

[1.2.6 CD-Spectroscopy 4](#_Toc201655348)

[1.2.7 Fluorescence Spectroscopy 4](#_Toc201655349)

[1.2.8 Irradiation Setup 4](#_Toc201655350)

[1.2.9 Dynamic Light Scattering (DLS) 4](#_Toc201655351)

[1.2.10 Matrix-Assisted Laser Desorption/Ionisation - Time of Flight Mass Spectrometry (MALDI-TOF-MS) 4](#_Toc201655352)

[1.2.11 2D Cell Culture 5](#_Toc201655353)

[1.2.12 Luminescence Detection of Cell Viability 5](#_Toc201655354)

[1.2.13 Confocal Laser Scanning Microscopy (CLSM) 5](#_Toc201655355)

[1.2.14 Fluorescence Phase Contrast Microscopy 6](#_Toc201655356)

[1.2.15 UV/Vis Spectroscopy 6](#_Toc201655357)

[2. Synthesis and Structural Characterization 7](#_Toc201655358)

[2.1 Synthesis of Compounds **1** and **2** 7](#_Toc201655359)

[2.2 TEM Analysis of **1** in different buffers 24](#_Toc201655360)

[2.3 TEM Analysis of incubation of **2** with GSH 25](#_Toc201655361)

[2.4 Determination of CAC 29](#_Toc201655362)

[2.5 Chemical Design of Coassembly Approach 30](#_Toc201655363)

[2.6 Coassembly Analysis 31](#_Toc201655364)

[2.7 ^1^H-NMR analysis of **1** with light switching 32](#_Toc201655365)

[2.8 LCMS Kinetic 33](#_Toc201655366)

[2.9 Confocal Laser Scanning microscopy of 2 at various concentrations 35](#_Toc201655367)

[References 35](#_Toc201655368)

# General Information

## Materials

Reagents and solvents were purchased from commercial sources and were used without further purification. Peptide synthesis grade reagents were used for synthesizing the peptides. HPLC was performed using acetonitrile (CH_3_CN) in HPLC grade and water obtained from a Millipore purification system. Flash column chromatography was carried out using Macherey-Nagel silica gel 0.04–0.063 mm.

## Instruments

### 1.2.1 Nuclear Magnetic Resonance (NMR) Spectroscopy

NMR spectra of small molecules and peptides were recorded on a Bruker Avance III 700 MHz spectrometer and a Bruker Avance 400 MHz spectrometer. The solvent signal was used as a reference (deuterated chloroform CDCl_3_ *δ*=7.26 ppm for ^1^H, *δ*=77.16 ppm for ^13^C, DMSO-d_6_ *δ*=2.50 ppm for ^1^H and *δ*=39.52 ppm for ^13^C). All ^1^H resonances are reported to the nearest 0.01 ppm. The multiplicity of ^1^H signals are indicated as: s = singlet; d = doublet; t = triplet; q = quartet; p = pentet; m = multiplet; br = broad; or combinations of thereof. Coupling constants (*J*) are quoted in Hz and reported to the nearest 0.1 Hz. Where appropriate, averages of the signals from peaks displaying multiplicity were used to calculate the value of the coupling constant. Chemical shifts for protons of the compounds were assigned on the basis of COSY and HSQC correlations. The data was processed in MestReNova.

### 1.2.2 Liquid Chromatography-Mass Spectrometry (LCMS)

Compounds were analyzed by HPLC-ESI-MS on a LC-MS 2020 by Shimadzu using a Kinetex 2.6 μm EVO C18 100 Å LC 50 × 2.1 mm column. MilliQ water acidified with 0.1% formic acid and CH_3_CN were used as solvents for all measurements. The solvent gradient started with 5% CH_3_CN and 95% water. This solvent ratio was kept constant for 2 min, then the CH_3_CN content was linearly increased to 95% in 14 min. Data were processed in LabSolutions and Origin 2024b.

### 1.2.3 Transmission Electron Microscopy (TEM)

TEM images of the peptide samples were recorded on a JEOL 1400 transmission electron microscope at a voltage of 120 kV. Formvar/carbon-film coated copper grids (300 mesh) by Plano GmbH were used to prepare the samples. The images were processed in Fiji ImageJ.

### 1.2.4 Microwave Peptide Synthesizer

Peptides were synthesized in a Liberty Blue Automated Microwave Peptide Synthesizer by CEM Corporation.

### 1.2.5 High-Performance Liquid Chromatography (HPLC)

The synthesized peptides were purified by preparative HPLC using a Shimadzu setup. A YMC-Actus Triart C18 column (150 x 20 mm, 5 μm) with a flowrate of 20 mL/min was used for purification. For analytical measurements an Atlantis T3 column (4.6 × 100 mm, 5 μm) was used at a flowrate of 1 mL/min. All purification steps were performed by using gradients of MilliQ water and acetonitrile, each acidified with 0.1% formic acid. Analytical measurements were performed by using gradients of MilliQ water and acetonitrile, each acidified with 0.1% trifluoroacetic acid. Absorbance was recorded at 214, 254, and 400 nm wavelength. The HPLC data were processed with the software LabSolutions by Shimadzu and Origin 2024b by OriginLab.

### 1.2.6 CD-Spectroscopy

CD spectra of the peptides were recorded on a JASCO J-1500 spectrometer in a 0.1 cm High Precision Cell by HellmaAnalytics. The recorded data were processed in Spectra Analysis by JASCO and Origin 2024b by OriginLab.

### 1.2.7 Fluorescence Spectroscopy

To record the fluorescence intensity, a SPARK 20M microplate reader by Tecan Group Ltd. was used. The samples were measured in a Greiner 384 flat black well plate and data was processed in Origin 2024b by OriginLab.

### 1.2.8 Irradiation Setup

Irradiations were performed either with a Thorlabs M365L3 lamp with a nominal wavelength of 365 nm, a bandwith (FWHM) of 9 nm and a current of 1000 mA or with a Avonec 3 W Power LED with a wavelength of 515-525 nm, a current of 750 mA and a voltage of 3.5-4.5 V.

### 1.2.9 Dynamic Light Scattering (DLS)

Single-angle DLS measurements were performed at 25 °C using a Malvern ZetaSizer Nano S purchased from Malvern Instruments Ltd. (Malvern, Great Britain) with a He/Ne Laser (λ = 633 nm) at a fixed scattering angle of 173°. All measurements were performed in triplicate. The obtained data was processed by cumulant fitting for D_h_.

### 1.2.10 Matrix-Assisted Laser Desorption/Ionisation - Time of Flight Mass Spectrometry (MALDI-TOF-MS)

MALDI-TOF spectra were recorded on either a rapifleX MALDI-TOF/TOF from Bruker or MALDI Synapt G2-SI from Waters. Samples were mixed with a saturated solution of the matrix S7 α-cyano-4-hydroxycinnamic acid (CHCA) in NH_4_HCO_3_ buffer (20 mM)/CH_3_CN 1/1. Data processing was performed in mMass.

### 1.2.11 2D Cell Culture

A549 cells were cultured at 37 °C and 5% CO_2_ in Dulbecco’s Modified Eagle’s Medium (DMEM, high glucose), supplemented with 10% fetal bovine serum (FBS). The cells were cultured in T75 culture flask and subcultivated two to three times per week.

### 1.2.12 Luminescence Detection of Cell Viability

A549 lung carcinoma cells were seeded into two 96-well half-area white plates (Greiner Bio-One, Cat# 675083) at a density of 2,500 cells per well in 50 µL of DMEM (Gibco, Cat# 11965092) supplemented with 10% FBS (Gibco, Cat# A5256801). Cells adhered overnight at 37°C in a humidified incubator with 5% CO₂.

Following incubation, the culture medium was carefully replaced with compound working solutions, prepared in DMEM with 10% FBS, including either **2*_trans_*** or **2*_cis_*** at concentrations ranging from 1 µM to 500 µM. Each solution contained 1% DMSO. Control wells received medium containing no compounds. Cells were exposed to the respective treatments for 4 hours.

Cell viability was assessed using the CellTiter-Glo Assay (Promega, Cat# G7571), which quantifies intracellular ATP as a reference for metabolically active cells. The assay relies on a thermostable luciferase that catalyzes the mono-oxygenation of luciferin to oxyluciferin in the presence of ATP, Mg²⁺, and O₂, producing a luminescent signal proportional to ATP levels.

50 µL of CellTiter-Glo reagent was added to each well. Plates were shaken gently for 2 minutes on an orbital shaker in the dark, followed by a period of incubation at room temperature without agitation, also shielded from light. Luminescence was recorded using a Tecan Spark 20M spectrophotometer. Data were processed in Microsoft Excel 2016 and graphs were generated in Origin 2024b.

### 1.2.13 Confocal Laser Scanning Microscopy (CLSM)

Cells were cultured in an ibidi µ-Slide 8 well at a density of 20,000 cells per well in DMEM and allowed to adhere overnight at 37°C and 5% CO_2_. The medium was aspirated and samples were introduced to each well after dissolving them in DMEM at the respective concentrations. Incubation was performed at 37°C and 5% CO_2_. Finally, cells were co-stained with MitoTracker Orange CMTMRos (Thermo Fisher Scientific).

Cells were imaged under physiological conditions using an incubator-equipped Leica Stellaris^®^ 8 microscope (40x glycerol objective, HC PL APO CS2 40x/1.25 GLYC) with fast lifetime contrast (FALCON) module (Leica Microsystem GmbH). The incubator (okolab) is set and held constant at 37°C, 5% CO_2_ and a relative humidity of 90% throughout all measurements. Samples were exited using a 40 MHz pulsed white light laser tuned to 649 nm for intensity measurements. Emitted photons were detected using HyD^®^ R detector with a filter window at 658-834 nm. Imaging was conducted with living cells with a scanning resolution of 1024 x 1024 pixels at 400 Hz for each image.

*Experimental Preparation*

A549 cells were seeded at a density of 20,000 cells per well in an 8-well chamber imaging slide. After adhering for 24 h, cells were treated with the sample for 4 h at 37°C. The samples were dissolved in DMSO at a concentration of 10 mM. Sample solutions were further diluted to an end concentration of 25, 50, 100 and 200 µM with DMEM and added to the cells (total DMSO content = 1.00 %). Cells were then co-stained with MitoTracker® Orange CMTMRos (Thermo Fisher Scientific) for 10 min.

### 1.2.14 Fluorescence Phase Contrast Microscopy

A 6-well plate was used to culture cells at a density of 80,000 cells per well in DMEM. After cells adhered overnight at 37 °C and 5% CO_2_, the medium was replaced by samples after dissolving them in DMEM at the respective concentrations. Incubation was also performed at 37°C and 5% CO_2_. Finally, cells were co-stained with MitoTracker Orange CMTMRos (Thermo Fisher Scientific).

Cells were imaged using a KEYENCE BZ-X810 microscope with a CCD-camera (Plan Fluorit 20x LD PH, NA 0.45 WD 8.8-7.5 mm Phase Contrast). Samples were illuminated using a 40 W LED light and using a Cy5 fluorescence filter cube (Ex: 620/60, Em: 700/75 nm).

*Experimental Preparation*

A549 cells were seeded at a density of 80,000 cells per well in an 6-well plate. After adhering for 24 h, cells were treated with the sample for 4 h at 37 °C. The samples were dissolved in DMSO at a concentration of 10 mM. Sample solutions were further diluted to an end concentration of 100 µM with DMEM and added to the cells (total DMSO content = 1.00 %). Cells were then co-stained with MitoTracker® Orange CMTMRos (Thermo Fisher Scientific) for 10 min.

### 1.2.15 UV/Vis Spectroscopy

UV/vis absorption spectra were recorded with a JASCO V-770 double-beam spectrophotometer at 20 °C using a 10 x 10 mm quartz cuvette by HellmaAnalytics. The recorded data were processed in Origin 2024b by OriginLab.

# 2. Synthesis and Structural Characterization

## 2.1 Synthesis of Compounds **1** and **2**

Figure S1: Synthesis of compounds **1** and **2**. **A)** Synthesis of **2**. (i) 20% Piperidine in DMF, 2x 10 min, RT. (ii) Oxyma, DIC, Fmoc-Ser(OtBu)-OH. (iii) Oxyma, DIC, Fmoc-Ile-OH. (iv) Oxyma, DIC, AAP. (v) TFA (95%), TIPS (2.5%), H_2_O (2.5%). **B)** Synthesis of **1-TAT**. (vi) 2,2’-dipyridyldisulfide, MeOH, AcOH, 24h, RT. (vii)4-nitrophenyl chloroformate, TEA, THF, 2 h, 0°C – RT. (viii) H_2_N-Ser(OH)-OtBu, DIPEA, DCM, overnight, RT. (ix)TFA(50%) in DCM, 2 h, RT. (x) DIC, DMAP, DCM, overnight, RT. (xi) Fmoc-Ala-OH, PyBOP, DIPEA, overnight, RT. (xii) CysTAT, MeOH, overnight, RT.

The synthesis of the linear tripeptide **1** was carried out using Fmoc solid-phase peptide synthesis on an alanine-preloaded Wang resin using *N,N’*-diisopropylcarbodiimide (DIC) and ethyl (2*Z*)-2-cyano-2-(hydroxyimino)acetate (OxymaPure) for the iterative coupling steps (Figure S1A). The photoswitch carboxylic acid was synthesized in a four-step reaction sequence starting from aniline as described in a previous report.^[50]^ It was then coupled to the ISA peptide on the solid support using DIC and OxymaPure, cleaved off from the resin with trifluoroacetic acid (TFA):triisopropyl silane (TIPS):H_2_O (95:2.5:2.5) and purified by high-performance liquid chromatography (HPLC) to yield **1**.

The synthesis of the photoswitchable isotripeptide **2** was carried out in solution (Figure S1B). The AAP photoswitch was attached to the *N*-terminus of isoleucine using DIC and OxymaPure, while a disulfide linker prepared from 2-mercaptoethanol was conjugated on the *N*-terminus of serine. Then, both fragments were combined *via* a Steglich esterification. The *C*-terminus was prolonged by alanine before the 2-mercaptopyridine substituent was replaced *via* a disulfide exchange reaction with Cys-TAT, yielding the bioactive molecule **2**, which was characterized by matrix-assisted laser desorption/ ionization mass spectrometry (MALDI) and HPLC (Figure S20).

The Wang resin preloaded with Fmoc-Ala (0.5 mmol) was swollen in 5 mL DMF at room temperature for 1 h before transferring it into the peptide synthesizer. DMF was removed via a draining process and the resin swollen with 20 mL DMF for 20 s. After draining of the DMF, the Fmoc group was removed with 10 mL 20 % v/v piperidine in DMF and a heating cycle at 70°C for 25 s followed by another cycle at 90°C for 65 s. The solution was drained, and the resin washed with DMF three times. After the last washing step, Fmoc-Ser(tBu)-OH (0.2 M in DMF, 10 mL), DIC solution (0.5 M in DMF, 4 mL) and OxymaPure solution (1.0 M in DMF, 2 mL) was added to the resin and the solution heated to 70°C for 30 s followed by a heating cycle to 90°C for 120 s. The solution was drained, the Fmoc group removed as described before and washed with DMF three times. After the last washing step, Fmoc-Ile-OH (0.2 M in DMF, 10 mL), DIC solution (0.5 M in DMF, 4 mL) and OxymaPure solution (1.0 M in DMF, 2 mL) was added to the resin and the solution heated to 70°C for 30 s followed by a heating cycle to 90°C for 120 s. The solution was drained, and the resin washed with DMF three times.

The AAP photoswitch (171 mg, 0.66 mmol, 1.3 eq) was then coupled to the free *N*-terminus by addition of DIC (189 mg, 1.5 mmol, 3 eq) and Oxyma (426 mg, 3 mmol, 6 eq) in DMF (10 mL) by shaking at room temperature overnight. The resin was then washed with DMF (10 mL, 3x) and DCM (10 mL, 3x), drained and cleaved from the resin by adding 10 mL of cleavage cocktail (95% TFA, 2.5% TIPS, 2.5% MilliQ water) to the resin and shaking at room temperature for 2 h. The cleavage cocktail was drained and the liquids removed under reduced pressure. The peptide was purified via HPLC with a YMC-Actus Triart C18 column (flowrate 20 mL/min). After lyophilisation, the product Fmoc-ICA was received as a yellow solid (85 mg, 0.16 mmol, 32%).


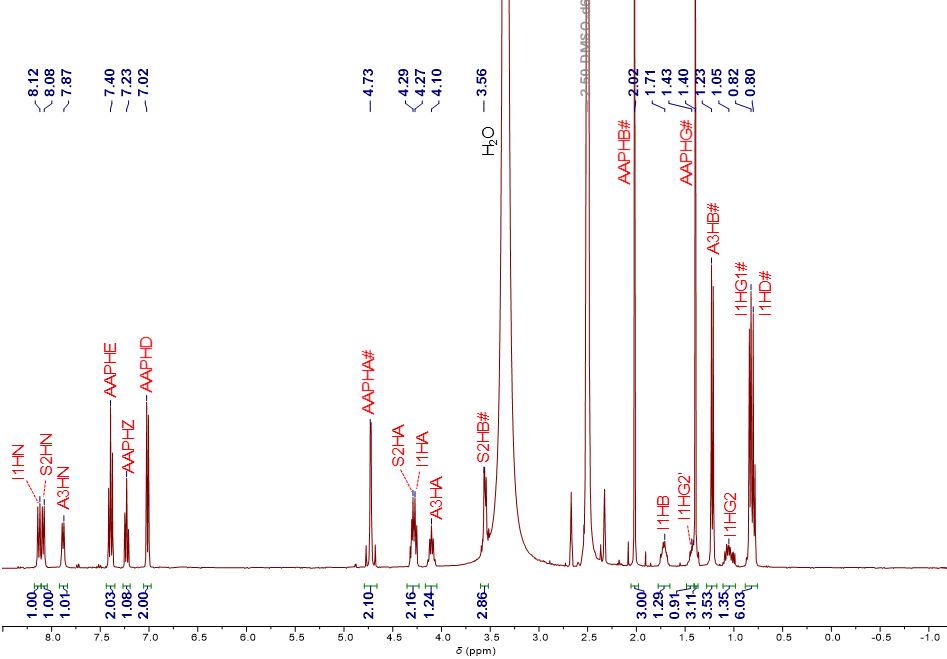


Figure S2: ^1^H-NMR (400 MHz, DMSO-d_6_, 298 K) of **1*_cis_***. Peak Assignments were made with comparison of subsequent 2D-NMR-spectra. ^1^H NMR (400 MHz, DMSO-d_6_) δ = 8.12 (d, 1H), 8.08 (d, 1H), 7.87 (d, 1H), 7.40 (t, 2H), 7.23 (tt, 1H), 7.02 (dd, 2H), 4.71 (q, 2H), 4.35 – 4.22 (m, 2H), 4.10 (p, 1H), 3.55 (dd, 2H), 2.02 (s, 3H), 1.78 – 1.65 (m, 1H), 1.49 – 1.41 (m, 1H), 1.40 (s, 3H), 1.22 (d, 3H), 1.11 – 0.97 (m, 1H), 0.89 – 0.73 (m, 6H).

Figure S3: ^1^H,^1^H-COSY NMR spectrum (400, 400 MHz, DMSO-d_6_, 298 K) of **1*_cis_***. ^1^H-^1^H NMR ((400, 400) MHz, DMSO-d_6_) δ (8.14 4.27), (8.14 8.11), (8.09 4.27), (8.09 8.05), (7.89 7.87), (7.89 4.09), (7.41 7.01), (7.39 7.20), (7.39 7.38), (7.24 7.38), (7.24 7.01), (7.22 7.20), (7.02 7.38), (7.02 7.01), (4.73 4.73), (4.30 8.08), (4.29 3.54), (4.28 1.71), (4.27 4.27), (4.27 8.13), (4.10 1.21), (4.10 4.09), (4.10 7.88), (3.56 3.54), (3.55 4.27), (2.01 2.01), (1.72 4.27), (1.72 0.79), (1.71 1.04), (1.71 1.40), (1.71 1.71), (1.41 0.79), (1.41 1.04), (1.40 1.71), (1.39 1.40), (1.23 1.22), (1.23 4.09), (1.06 1.71), (1.05 0.79), (1.05 1.04), (1.04 1.40), (0.83 1.70), (0.82 0.79), (0.80 1.04), (0.80 1.40), (0.79 0.79).


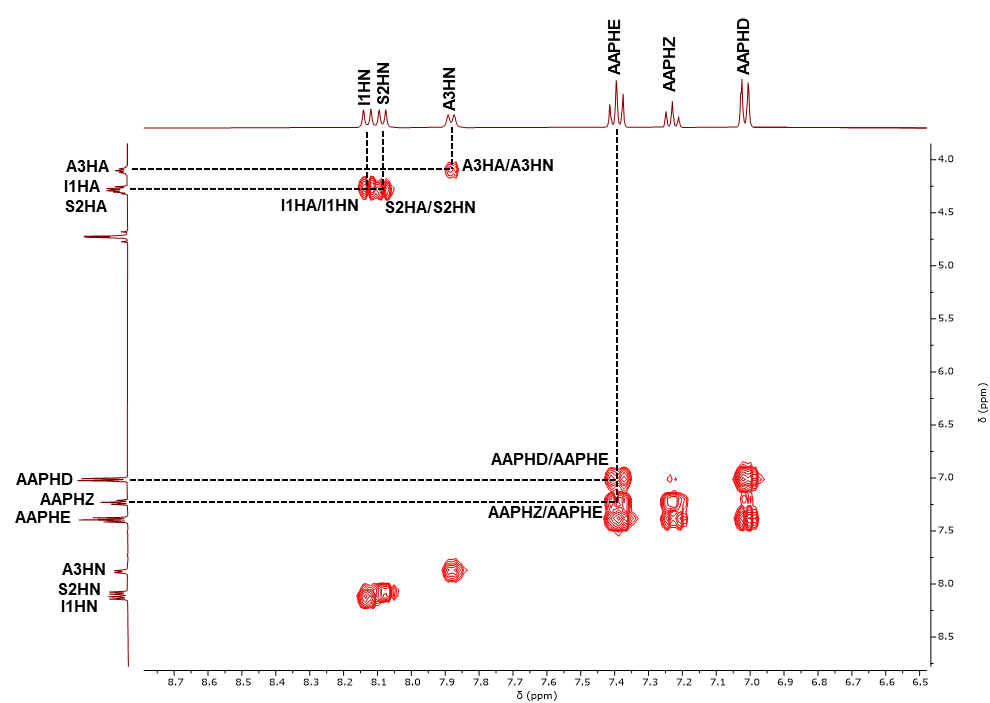


Figure S4: ^1^H,^1^H-COSY NMR spectrum (400, 400 MHz, DMSO-d_6_, 298 K) of **1*_cis_***. Zoom of fingerprint region relevant for interactions of the amide protons with the α-protons of the amino acids.


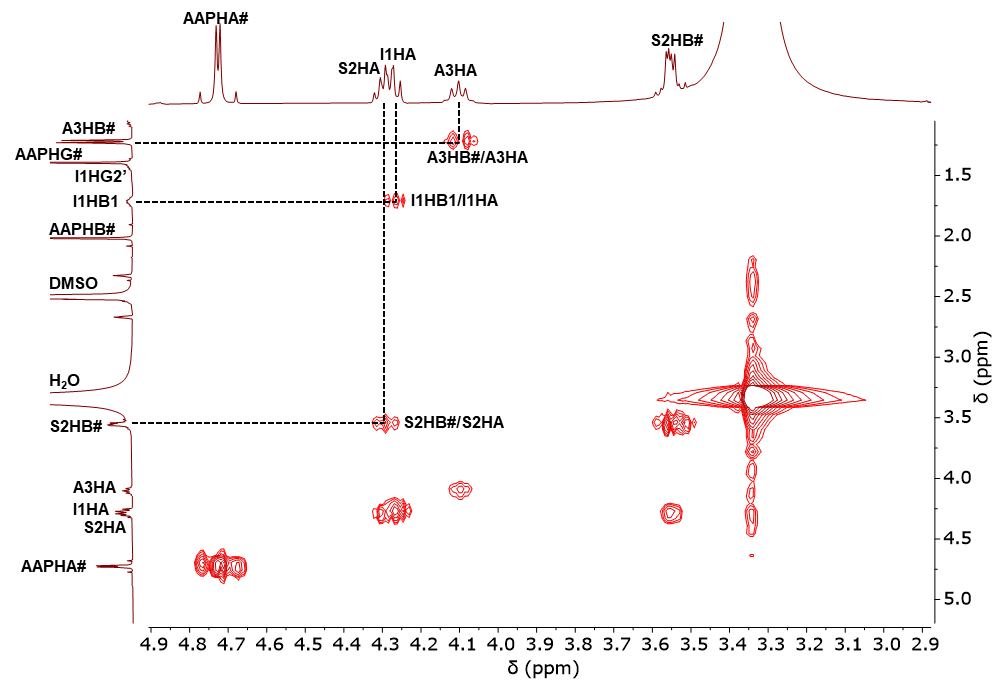


Figure S5: ^1^H,^1^H-COSY NMR spectrum (400, 400 MHz, DMSO-d_6_, 298 K) of **1*_cis_***. Zoom of region with the chemical shift of 5–3 ppm, 5–1 ppm.
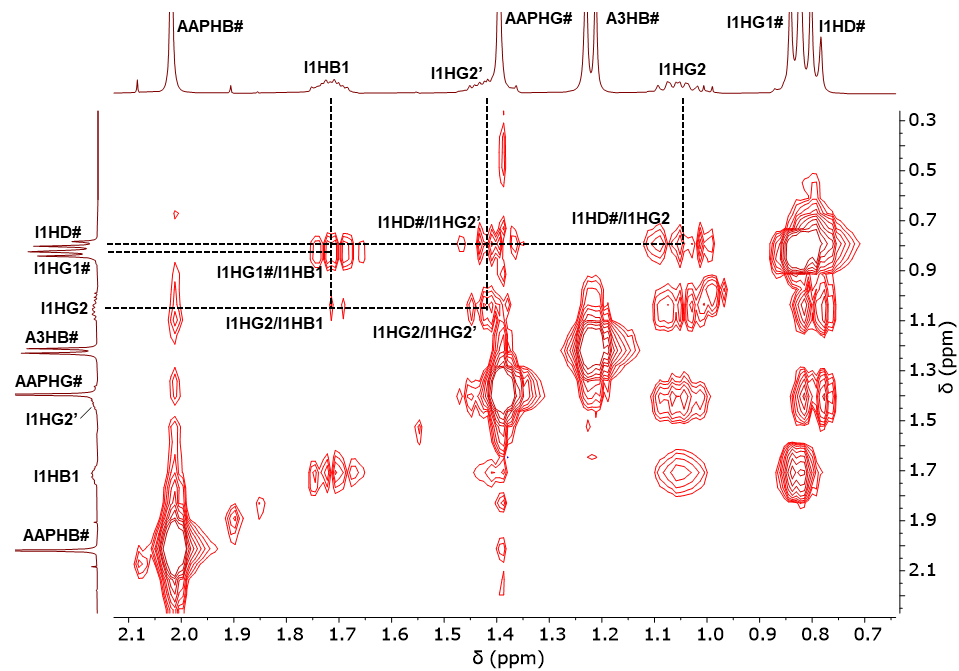


Figure S6: ^1^H,^1^H-COSY NMR spectrum (400, 400 MHz, DMSO-d_6_, 298 K) of **1*_cis_***. Zoom of region with the chemical shift of 2–0.5 ppm, 2–0 ppm.

Figure S7: ^1^H,^13^C-HSQC NMR spectrum (400, 101 MHz, DMSO-d_6_, 298 K) of **1*_cis_***. Red colour indicates CH/CH_3_ and blue colour indicates CH_2_. ^1^H-^13^C NMR ((400, 101) MHz, DMSO-d_6_) δ (7.40 129.35), (7.23 127.07), (7.01 119.68), (4.73 51.30), (4.29 54.99), (4.28 56.92), (4.10 47.96), (3.55 61.67), (2.02 9.46), (1.71 37.23), (1.44 23.87), (1.40 12.80), (1.22 17.48), (1.06 24.40), (0.82 15.26), (0.81 11.22).

Figure S8: ^1^H,^13^C-HMBC NMR spectrum (400, 101 MHz, DMSO-d_6_, 298 K) of **1*_cis_***. ^1^H-^13^C NMR ((400, 101) MHz, DMSO-d_6_) δ (7.24 119.92), (7.02 127.18), (4.72 136.09), (4.72 166.09), (2.17 9.52), (2.02 134.21), (2.02 136.09), (1.85 9.52), (1.55 12.80), (1.40 134.45), (1.40 136.09), (1.23 12.80), (1.22 174.04), (1.21 48.19), (1.05 17.49), (0.98 15.38), (0.95 11.16), (0.83 24.28), (0.83 37.18), (0.83 56.87), (0.66 15.38), (0.63 11.39).


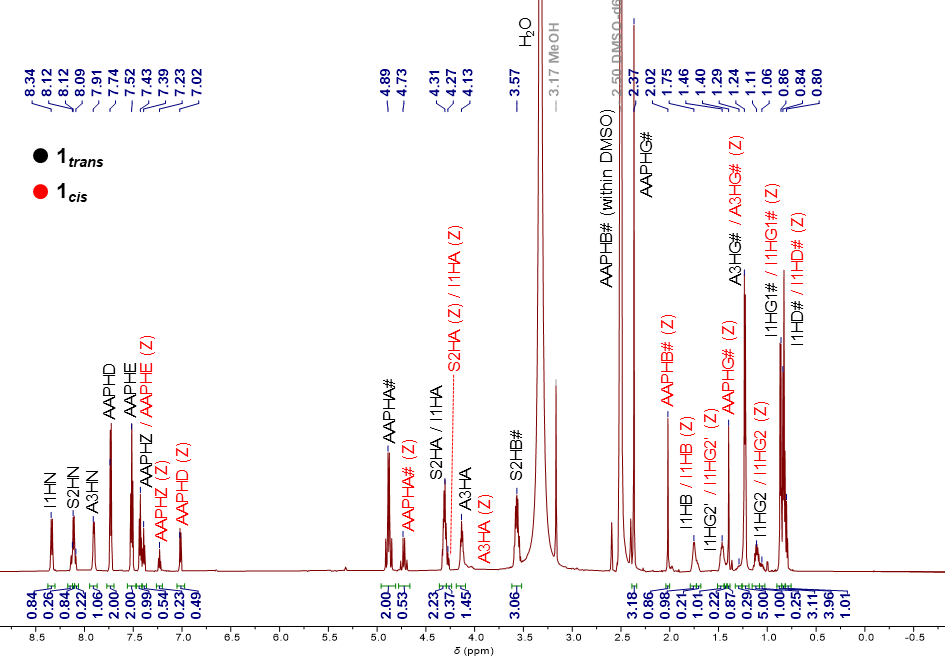


Figure S9: ^1^H-NMR (700 MHz, DMSO-d_6_, 298 K) of native **1*_trans_***. Peak Assignments were made with comparison of subsequent 2D-NMR-spectra. As described in the main text, the native state of compound **1** consists of approximately 85% **1*_trans_***.and 15% **1*_cis_***. The resulting peaks of both isomers are assigned with different colours: black = **1*_trans_***, red = **1*_cis_***. ^1^H NMR (700 MHz, DMSO-d_6_) δ = 8.34 (d, 1H), 8.12 (d, 1H), 8.11 (d, 0H), 7.91 (d, 1H), 7.74 (d, 2H), 7.52 (t, 2H), 7.43 (t, 1H), 7.39 (t, 1H), 7.23 (t, 0H), 7.02 (d, 0H), 4.89 (q, 2H), 4.72 (q, 1H), 4.36 – 4.25 (m, 3H), 4.18 – 4.08 (m, 2H), 3.64 – 3.49 (m, 3H), 2.37 (s, 3H), 2.02 (s, 1H), 1.79 – 1.70 (m, 1H), 1.51 – 1.44 (m, 1H), 1.40 (s, 1H), 1.23 (d, 5H), 1.16 – 1.08 (m, 1H), 0.90 – 0.76 (m, 8H).

Figure S10: ^1^H,^1^H-COSY NMR spectrum (700, 700 MHz, DMSO-d_6_, 298 K) of native **1*_trans_***. ^1^H-^1^H NMR ((700, 700) MHz, DMSO-d_6_) δ = (8.34 8.35), (8.34 4.31), (8.13 8.12), (8.12 4.31), (8.10 8.12), (8.10 4.31), (7.91 7.94), (7.91 4.13), (7.73 7.53), (7.72 7.76), (7.52 7.41), (7.51 7.71), (7.51 7.53), (7.44 7.71), (7.43 7.41), (7.40 7.00), (7.39 7.24), (7.39 7.41), (7.24 7.41), (7.23 7.24), (7.01 7.00), (7.01 7.41), (4.89 4.89), (4.72 4.72), (4.31 1.79), (4.31 8.12), (4.30 3.60), (4.30 4.31), (4.29 8.35), (4.14 1.26), (4.13 4.13), (4.13 7.94), (3.57 3.60), (3.56 4.31), (2.36 2.37), (2.02 2.02), (1.76 0.85), (1.76 1.49), (1.76 4.31), (1.75 1.14), (1.75 1.79), (1.46 0.85), (1.46 1.14), (1.46 1.49), (1.45 1.79), (1.23 4.13), (1.23 1.26), (1.10 0.85), (1.10 1.14), (1.10 1.49), (1.10 1.79), (0.87 0.85), (0.86 1.79), (0.84 1.49), (0.84 1.73), (0.83 0.85), (0.83 1.14).

Figure S11: ^1^H,^13^C-HSQC NMR spectrum (700, 176 MHz, DMSO-d_6_, 298 K) of native **1*_trans_***. ^1^H-^13^C NMR ((700, 176) MHz, DMSO-d_6_) δ = (7.74 121.11), (7.74 128.85), (7.52 128.85), (7.42 129.14), (7.24 126.80), (7.01 119.51), (4.90 51.05), (4.71 51.05), (4.33 54.70), (4.32 56.60), (4.12 47.70), (3.55 61.27), (2.50 9.16), (2.36 13.54), (2.01 9.31), (1.76 36.99), (1.46 23.88), (1.39 12.52), (1.23 28.57), (1.22 17.04), (1.09 23.90), (0.88 15.00), (0.85 10.77).

Figure S12: ^1^H,^13^C-HMBC NMR spectrum (700, 176 MHz, DMSO-d_6_, 298 K) of native **1*_trans_***. ^1^H-^13^C NMR ((700, 176) MHz, DMSO-d_6_) δ = (8.34 56.74), (8.33 165.92), (8.11 170.59), (8.11 54.99), (7.90 169.42), (7.74 121.26), (7.73 152.78), (7.51 129.14), (7.41 128.85), (7.40 119.80), (4.89 140.82), (4.88 165.92), (4.88 50.62), (4.73 136.40), (4.73 165.98), (4.32 165.92), (4.31 36.89), (4.31 170.30), (4.31 55.77), (4.30 24.05), (4.29 15.29), (3.57 54.70), (3.57 169.13), (2.37 134.39), (2.36 140.52), (2.02 135.85), (1.76 170.59), (1.75 24.05), (1.74 10.91), (1.73 15.29), (1.47 10.91), (1.45 56.74), (1.45 37.18), (1.41 133.87), (1.23 173.80), (1.23 47.69), (1.23 16.94), (1.11 36.89), (1.11 10.91), (1.10 55.81), (0.87 36.89), (0.86 16.94), (0.86 24.05), (0.86 56.74), (0.84 24.05), (0.83 36.89), (0.83 10.38).

Figure S13: ^13^C-NMR spectrum (176 MHz, DMSO-d_6_, 298 K) of native **1*_trans_***. ^13^C NMR (176 MHz, DMSO-d_6_) δ = 173.90, 170.60, 169.40, 166.08, 153.01, 141.00, 140.70, 134.52, 129.52, 129.18, 127.08, 121.39, 119.79, 61.65, 56.95, 55.02, 51.41, 47.99, 37.13, 17.40, 13.83, 11.29, 9.50.

In a round-bottom flask, H_2_N-Ile-OtBu x HCl (109 mg, 1.2 eq) was dissolved in dry DCM (5 mL). DIPEA (400 µL, 6 eq), DIC (146 mg, 3 eq), Oxyma (340 mg; 6 eq) and AAP (99 mg, 1 eq) were added and the solution was stirred at room temperature overnight. Water was added and the aqueous phase extracted with DCM twice. The combined organic phase was washed with 1 M HCl solution (2x) and brine (2x), dried over MgSO_4_ and the solvent was removed in vacuo. Subsequent silica gel column chromatography (cHex:EA 2:1) yielded an orange oil (133 mg, 0.31 mmol, 80%). The tBu protecting group of compound **4** was removed from the C-terminus by stirring in TFA/DCM (1:1) for 2 h at room temperature and removing the solvents in vacuo.


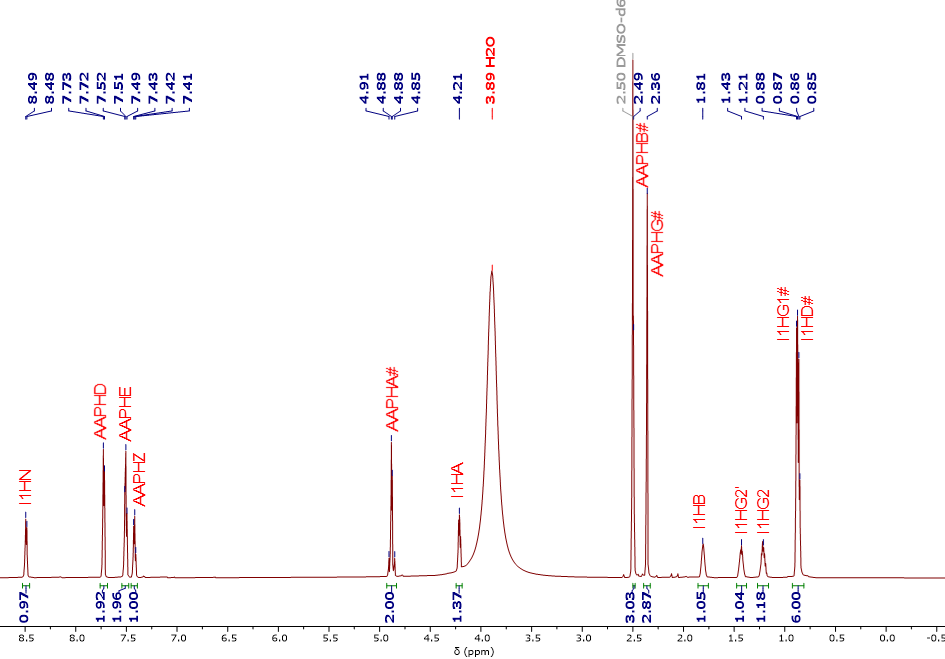


Figure S14: ^1^H-NMR (700 MHz, DMSO-d_6_, 298 K) of AAP-Ile-OH (**4**).

**^1^H NMR** (700 MHz, DMSO-d_6_, 298 K) δ 8.49 (d, *J* = 8.1 Hz, 1H), 7.72 (d, *J* = 7.2 Hz, 2H), 7.51 (t, *J* = 7.4 Hz, 2H), 7.42 (t, *J* = 7.2 Hz, 1H), 4.88 (q, *J* = 5.9 Hz, 2H), 4.23 – 4.19 (m, 1H), 2.49 (s, 3H, in solvent peak), 2.36 (s, 3H), 1.84 – 1.77 (m, 1H), 1.47 – 1.40 (m, 1H), 1.25 – 1.18 (m, 1H), 0.90 – 0.84 (m, 6H).

**^13^C NMR** (176 MHz, DMSO-d_6_, 298 K) δ 173.13, 166.87, 158.91, 158.70, 153.45, 141.59, 141.14, 134.99, 130.02, 129.67, 121.88, 116.53, 114.89, 56.93, 51.74, 40.43, 40.29, 40.17, 40.05, 39.93, 39.81, 39.69, 39.57, 37.01, 25.14, 16.02, 14.35, 11.80, 9.96.


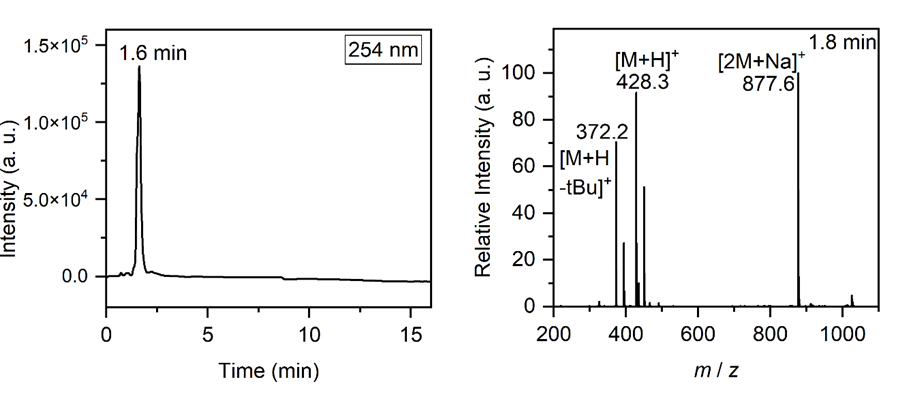


Figure S15: LC-MS data of AAP-Ile-tBu (**4**). Left: LC trace (254 nm) with t_R_ = 1.6 min. Right Convoluted ESI-MS spectrum showing peaks of [M+H]^+^ (calculated: 428.26), [2M+Na]^+^ (calculated: 877.51) and [M+H-tBu]^+^ (calculated: 372.20, protecting group removed in ESI-MS).

Compound **4** (31 mg, 1 eq) was dissolved in dry DCM (5 mL) and compound **5** (35 mg, 1.1 eq), DIC (25 µL, 2 eq) and DMAP (10 mg, 1 eq) were added. The reaction mixture was stirred at room temperature overnight. The solvent was reduced in vacuo and the residue purified via HPLC yielding a yellow solid (25 mg, 34.3 µmol, 43%).

**^1^H NMR** (700 MHz, DMSO-d_6_, 298 K) δ 8.62 (d, *J* = 8.8 Hz, 1H), 8.52 (d, J = 8.8 Hz, 1H), 8.44 (d, J = 4.7 Hz, 1H), 7.81 (t, J = 8.1 Hz, 2H), 7.76 (d, J = 7.8 Hz, 1H), 7.73 (d, J = 7.8 Hz, 2H), 7.51 (t, J = 7.7 Hz, 2H), 7.42 (t, J = 7.3 Hz, 1H), 7.23 (t, 1H), 4.96 – 4.82 (m, 2H), 4.55 (dd, J = 8.8 Hz, 1H), 4.37 – 4.27 (m, 3H), 4.27 – 4.21 (m, 1H), 4.19 (t, 2H), 3.07 (t, J = 6.3 Hz, 2H), 2.36 (s, 3H), 1.93 – 1.83 (m, 1H), 1.39 (s, 10H), 1.35 – 1.23 (m, 1H), 1.20 – 1.08 (m, 1H), 0.84 (d, J = 7.6 Hz, 6H).

**^13^C NMR** (176 MHz, DMSO-d_6_, 298 K) δ 171.26, 166.73, 158.85, 155.81, 153.02, 149.65, 140.95, 140.60, 137.89, 134.58, 129.61, 129.25, 121.46, 121.32, 119.32, 81.55, 64.01, 61.92, 55.00, 53.42, 51.28, 40.02, 39.88, 39.76, 39.64, 39.52, 39.40, 39.28, 39.16, 37.26, 36.58, 27.58, 25.66, 24.76, 15.39, 14.68, 13.92, 11.59, 11.24, 9.53.


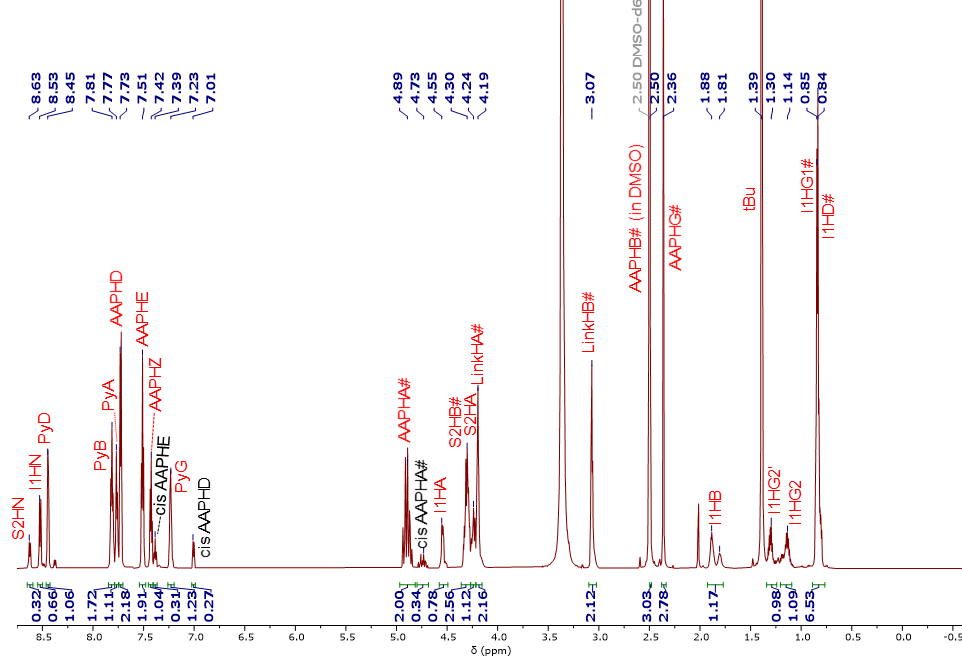


Figure S16: ^1^H-NMR (700 MHz, DMSO-d_6_, 298 K) of iso-AAP-Ile-Ser-tBu (**6**).


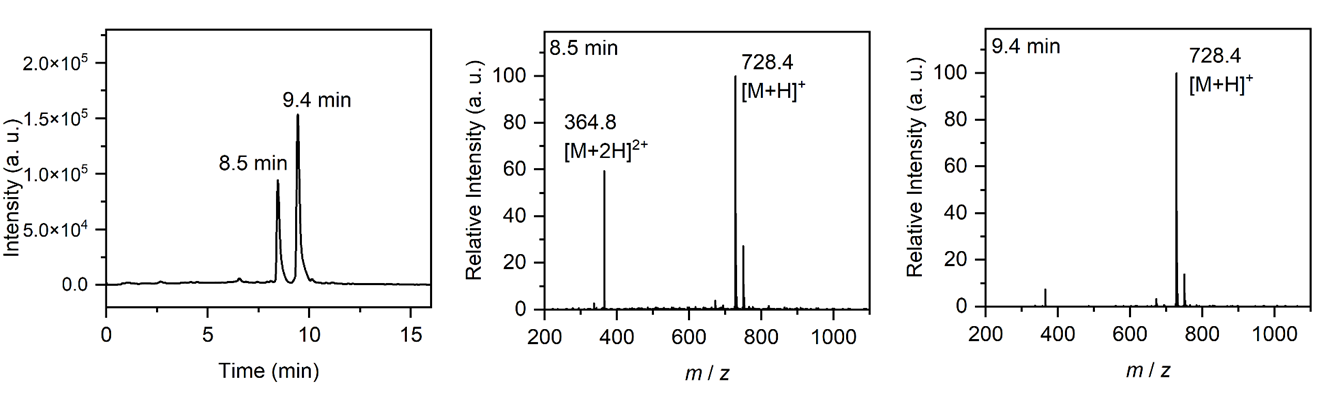


Figure S17: LC-MS data of **6**. Left: LC trace (254 nm) with t_R_ = 8.5 min and 9.4 min. Middle: Convoluted ESI-MS spectrum of *cis*-isomer showing peaks of [M+H]^+^ (calculated: 728.29) and [M+2H]^2+^ (calculated: 364.65). Right: Convoluted ESI-MS spectrum of *trans*-isomer showing peak of [M+H]^+^ (calculated: 728.29).

The tBu protecting group of compound **6** was removed from the *C*-terminus by stirring in TFA/DCM (1:1) for 2 h at room temperature. The solvents were removed in vacuo and, in a round-bottom flask, compound **6** (25 mg, 1 eq) was dissolved in dry DCM (5 mL) and DIPEA (40 µL, 6 eq), PyBOP (45 mg, 2.3 eq) and H_2_N-Ala-OtBu x HCl (13 mg, 1.9 eq) were added. The reaction mixture was stirred at room temperature overnight and the solvent removed in vacuo. The residue was purified by HPLC, stirred in TFA/DCM (1:1) for 2 h at room temperature and lyophilized, yielding a yellow solid (17 mg, 21.2 µmol, 59%).

**^1^H NMR** (700 MHz, DMSO-d_6_, 298 K) δ 8.62 (d, *J* = 8.2 Hz, 1H), 8.53 – 8.47 (m, 1H), 8.44 (d, *J* = 5.6 Hz, 1H), 8.37 (d, *J* = 7.2 Hz, 1H), 7.81 (t, *J* = 7.2 Hz, 1H), 7.79 – 7.75 (m, 1H), 7.73 (d, *J* = 7.7 Hz, 2H), 7.51 (t, *J* = 7.6 Hz, 2H), 7.47 – 7.36 (m, 1H), 7.27 – 7.19 (m, 1H), 4.96 – 4.82 (m, 2H), 4.49 (dd, *J* = 8.7, 4.4 Hz, 1H), 4.43 – 4.35 (m, 1H), 4.35 – 4.28 (m, 1H), 4.25 – 4.16 (m, 3H), 4.16 – 4.04 (m, 2H), 3.06 (t, *J* = 6.1 Hz, 2H), 2.37 (s, 3H), 1.93 – 1.79 (m, 1H), 1.38 (s, 9H), 1.33 – 1.27 (m, 1H), 1.25 (d, *J* = 7.2 Hz, 3H), 1.18 – 1.08 (m, 1H), 0.91 – 0.74 (m, 6H).

**^13^C NMR** (176 MHz, DMSO-d_6_) δ 171.90, 168.80, 167.19, 159.36, 156.13, 153.80, 150.07, 141.41, 138.32, 135.43, 129.95, 129.66, 121.87, 121.80, 120.15, 119.99, 81.03, 64.85, 62.36, 55.34, 53.86, 49.29, 37.66, 36.84, 33.34, 28.02, 26.05, 25.09, 17.77, 15.28, 14.34, 11.95, 11.70, 9.96.


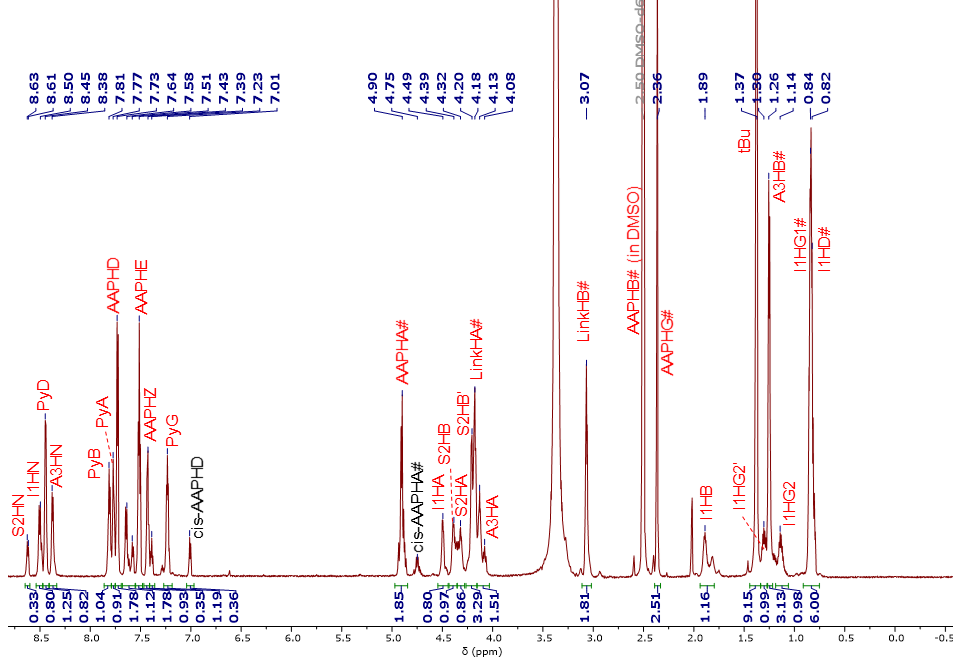


Figure S18: ^1^H-NMR (700 MHz, DMSO-d6, 298 K) of iso-AAP-Ile-Ser-Ala-OtBu (**7**).


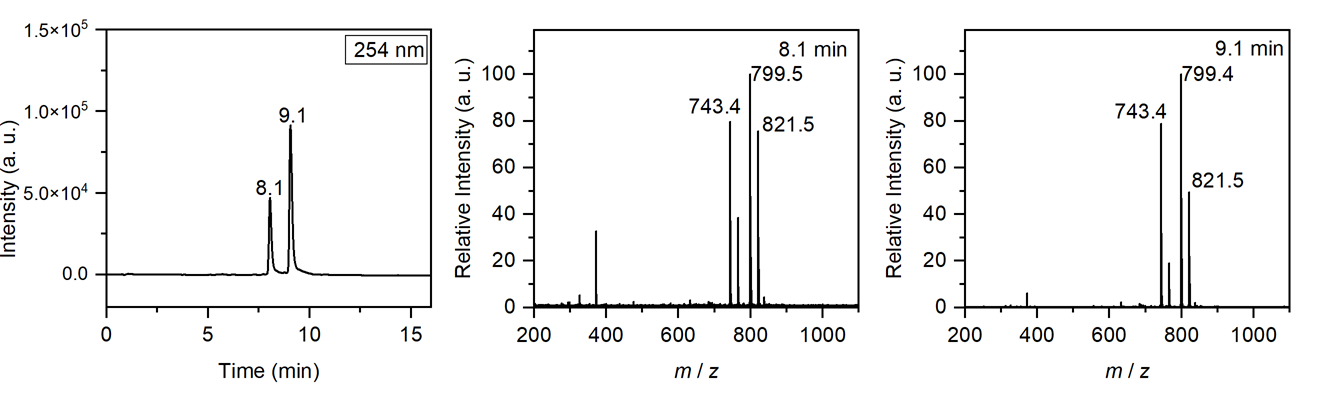


Figure S19: LC-MS data of compound **7**. Left: LC trace (254 nm) with t_R_ = 8.1 min and 9.1 min. Middle: Convoluted ESI-MS spectrum of *cis*-isomer showing peaks of [M-tBu+H]^+^ (calculated 743.26), [M+H]^+^ (calculated: 799.33) and [M+Na]^+^ (calculated: 821.31). Right: Convoluted ESI-MS spectrum of *trans*-isomer showing peaks of [M-tBu+H]^+^ (calculated 743.26), [M+H]^+^ (calculated: 799.33) and [M+Na]^+^ (calculated: 821.31).


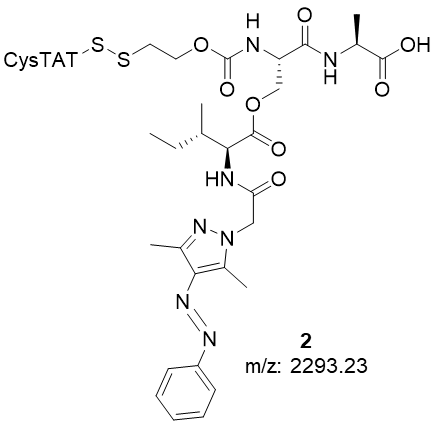


In a round-bottom flask compound **7** (1.4 mg, 1.8 µmol, 1 eq.) was dissolved in MeOH and CysTAT (5.0 mg, 3.0 µmol, 1.6 eq.) was added. The reaction mixture was stirred at room temperature overnight. The solvent was removed in vacuo and the peptide purified using HPLC. Subsequently, TFA/DCM (1:1) was added and the solution was stirred for 2 h at room temperature. The solvents were removed in vacuo and the sample was lyophilized, yielding a yellow solid (3 mg, 1.3 µmol, 72%).


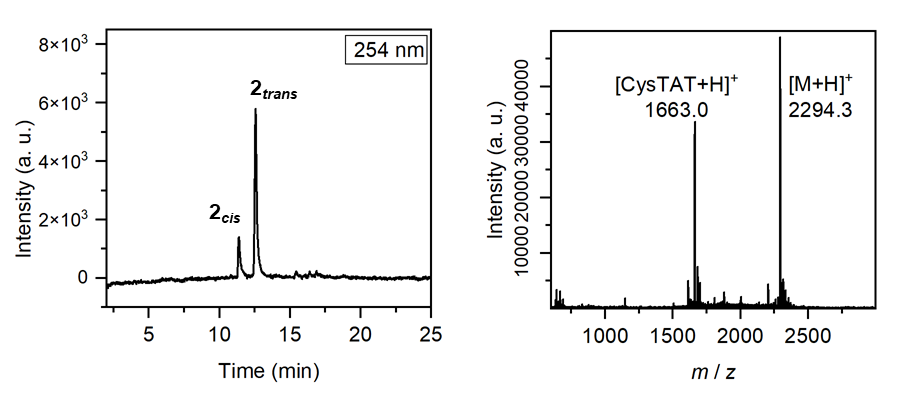


Figure S20: Left: HPLC trace (254 nm) of compound **2** with *t_R_* = 11.4 min (**2*_cis_***) and 12.6 min (**2*_trans_***). Right: Convoluted MALDI-MS spectrum of **2** showing peaks of [CysTAT+H]^+^ (calculated 1661.98, released due to strong ionization energy) and [M+H]^+^ (calculated: 2294.23).

Figure S21: Synthesis of **AAP**^1^. (i) HCl, NaNO_2_, HOAc, 45 min, 0°C. (ii) pentane-2,4-dione, NaOAc, EtOH/H_2_O, 1 h, RT. (iii) hydrazine, EtOH, 3 h, reflux. (iv) methyl 2-bromoacetate, K_2_CO_3_, ACN, 18 h, reflux. (v) LiOH, THF/H_2_O, overnight, RT.

The AAP photoswitch was synthesized according to a previously reported procedure.^1^

## 2.2 TEM Analysis of **1** in different buffers


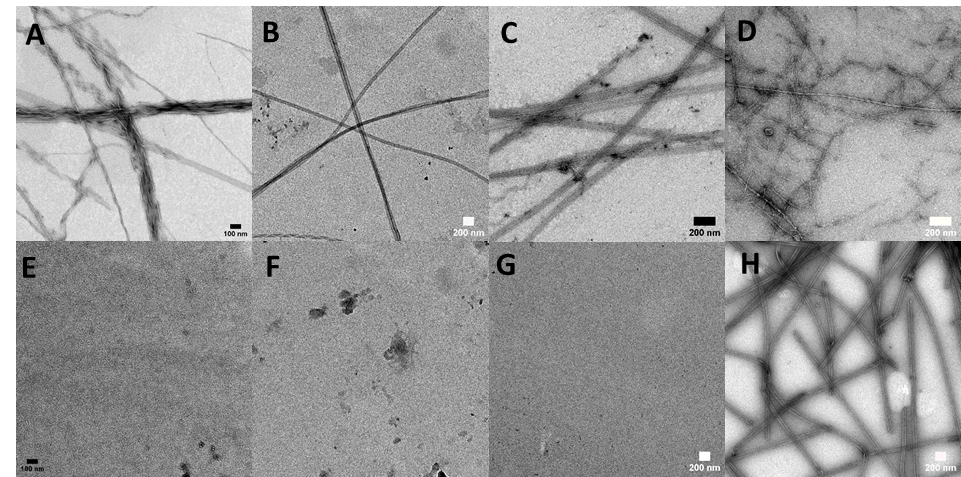


Figure S22: TEM images of **1**. A) **1*_trans_*,** 200 µM, HEPES buffer, 1% DMSO. B) **1*_trans_*,** 200 µM, PB buffer (10 mM Na_2_HPO_4_, 1.8 mM KH_2_PO_4_) with NaF (137 mM) and KF (2.7 mM). C) **1*_trans_*,** 200 µM, PBS, 1% DMSO. D) **1*_trans_*,** 150 µM, HEPES buffer, 10% DMSO, E) **1*_cis_*,** 200 µM, HEPES buffer, 1% DMSO, irradiated for 5 s at 365 nm. F) **1*_cis_*,** 200 µM, PB buffer (10 mM Na_2_HPO_4_, 1.8 mM KH_2_PO_4_) with NaF (137 mM) and KF (2.7 mM), irradiated for 5 s at 365 nm. G) **1*_cis_*,** 200 µM, PBS, 1% DMSO, irradiated for 5 s at 365 nm. H) **1*_trans_*,** 400 µM, PBS, 1% DMSO.

## 2.3 TEM Analysis of incubation of **2** with GSH


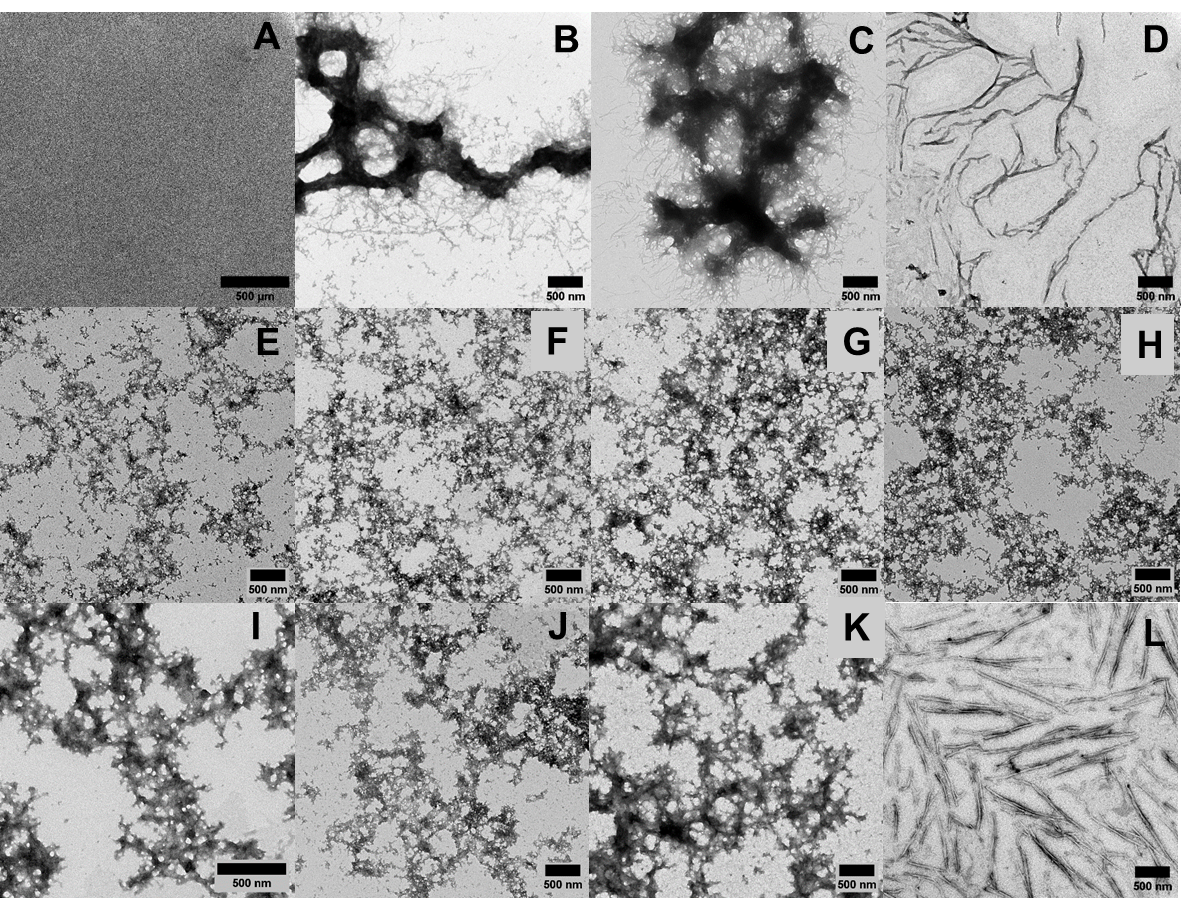
Figure S23: TEM images of **2** (200 µM, PBS) incubation with GSH (10 mM) over time. (A) **2*_trans_*** after 0 h. (B) **2*_trans_*** after 2 h. (C) **2*_trans_*** after 4 h. (D) **2*_trans_*** after 24 h. (E) **2*_cis_*** after 0 h. (F) **2*_cis_*** after 2 h. (G) **2*_cis_*** after 4 h. (H) **2*_cis_*** after 24 h. (I) **2*_trans_*** after 24 h without GSH. (J) **2*_cis_*** after 24 h without GSH. (K) Only GSH (10 mM) in PBS. (L) **2*_trans_*** after 3 d.


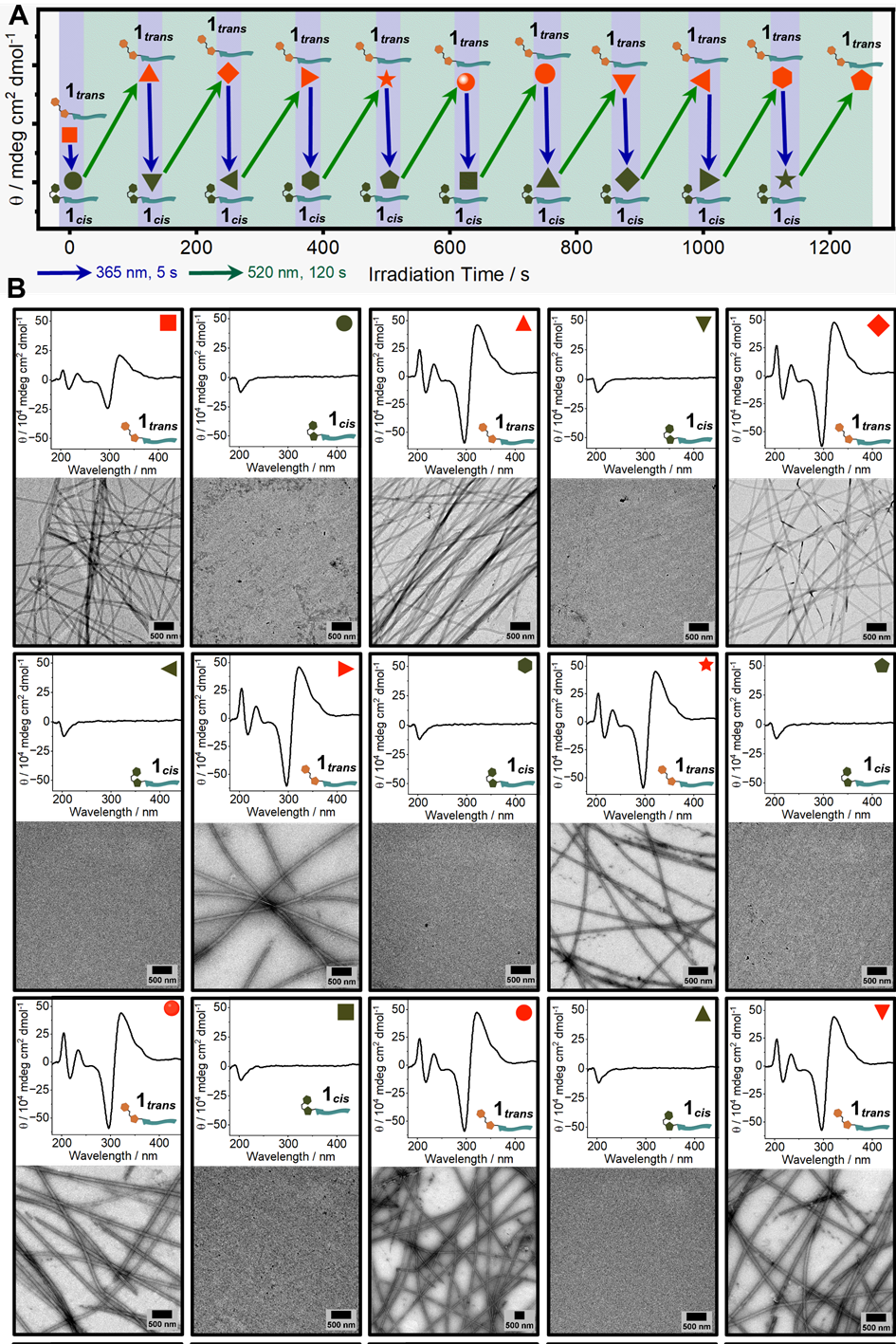


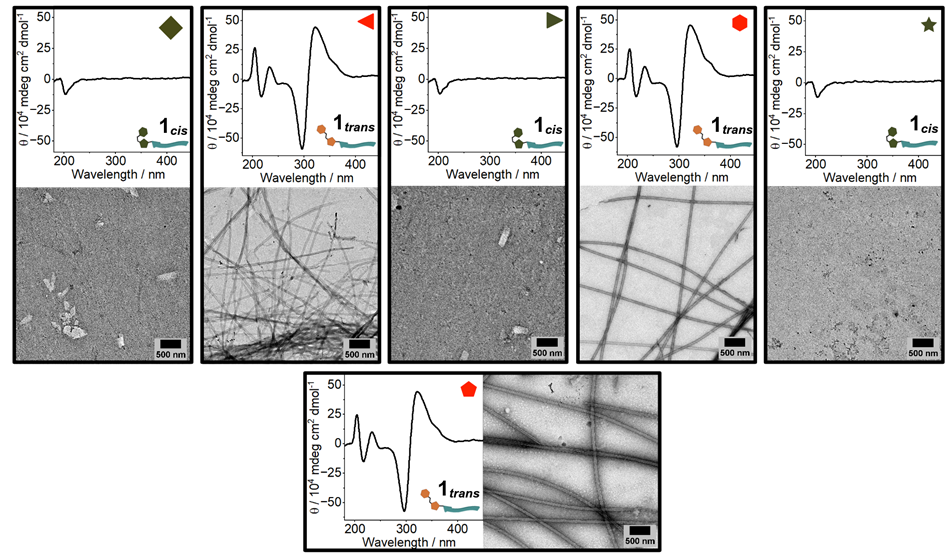


Figure S24: (**A**) Molar ellipticity of **1** at 325 nm over the course of 10 irradiation cycles at 365 nm for 5 s (blue box) and 520 nm for 120 s (green box). In between the individual irradiation steps, corresponding TEM micrographs and CD-spectra at 200 µM in PBS were recorded (**B**). Individual measurements are indicated by different symbol shape. Scale bar for TEM-micrographs = 500 nm.


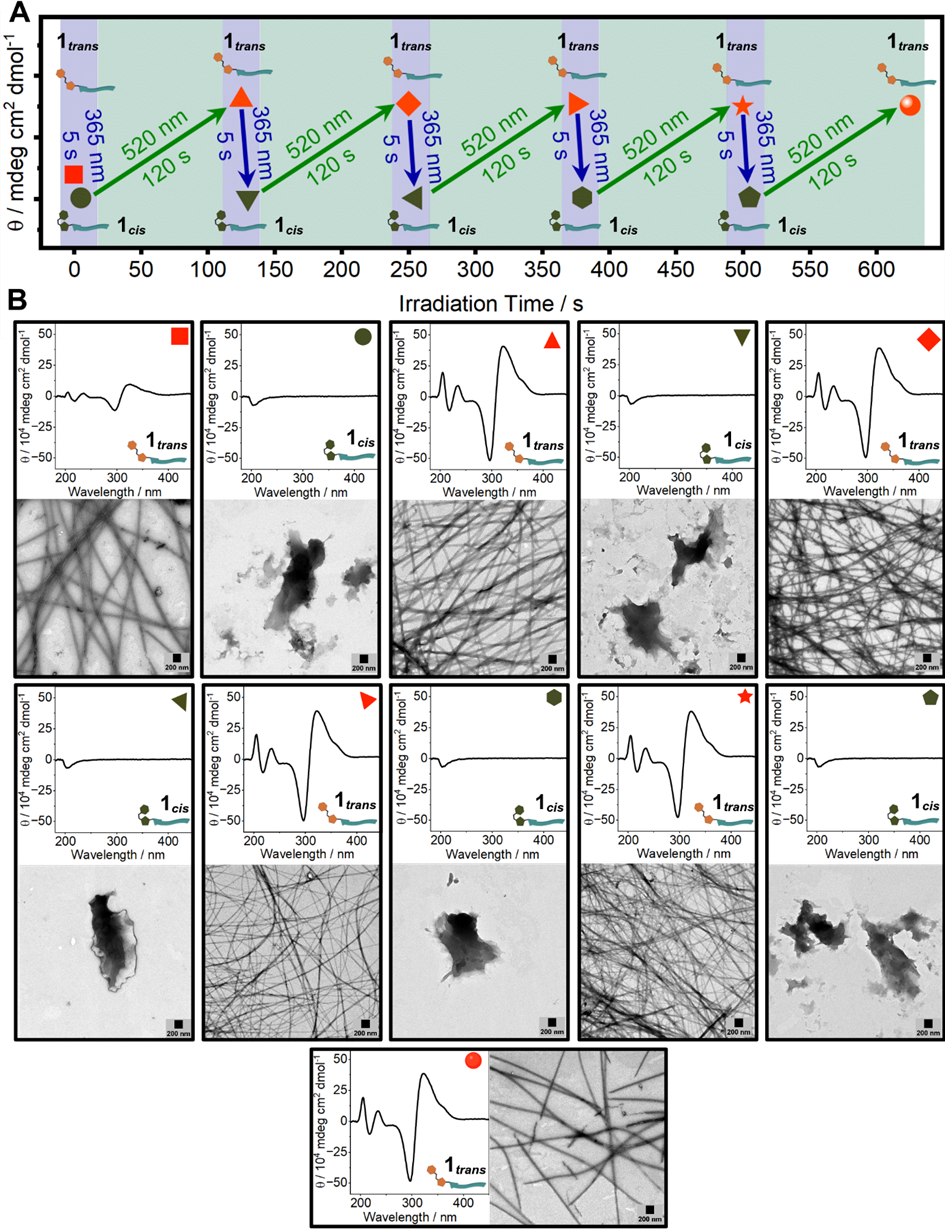


Figure S25: (**A**) Molar ellipticity of **1** at 325 nm over the course of 10 irradiation steps at 365 nm for 5 s (blue box) and 520 nm for 120 s (green box). Inbetween the irradiation steps, corresponding TEM micrographs and CD-spectra at 400 µM in PBS were recorded (**B**). Individual measurements are indicated by different symbol shape. Scale Bars = 200 nm.

## 2.4 Determination of CAC


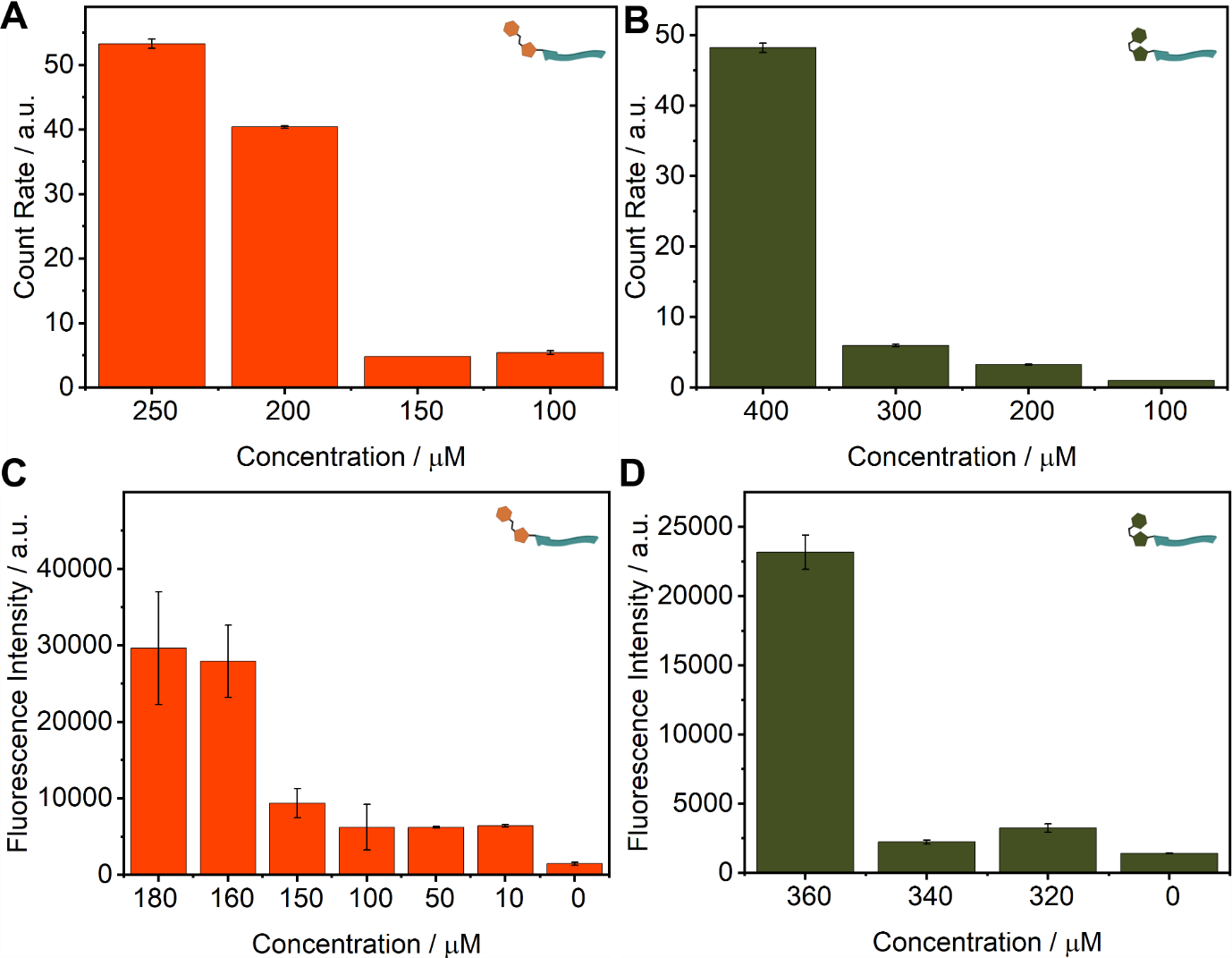


Figure S26: DLS count rate assay of **1*_trans_*** (**A**) and **1*_cis_*** (**B**) and Proteostat aggregation assay of **1*_trans_*** (**C**) and **1*_cis_*** (**D**). Measured in PBS with 1% DMSO. The data are presented as mean±SEM.

## 2.5 Chemical Design of Coassembly Approach


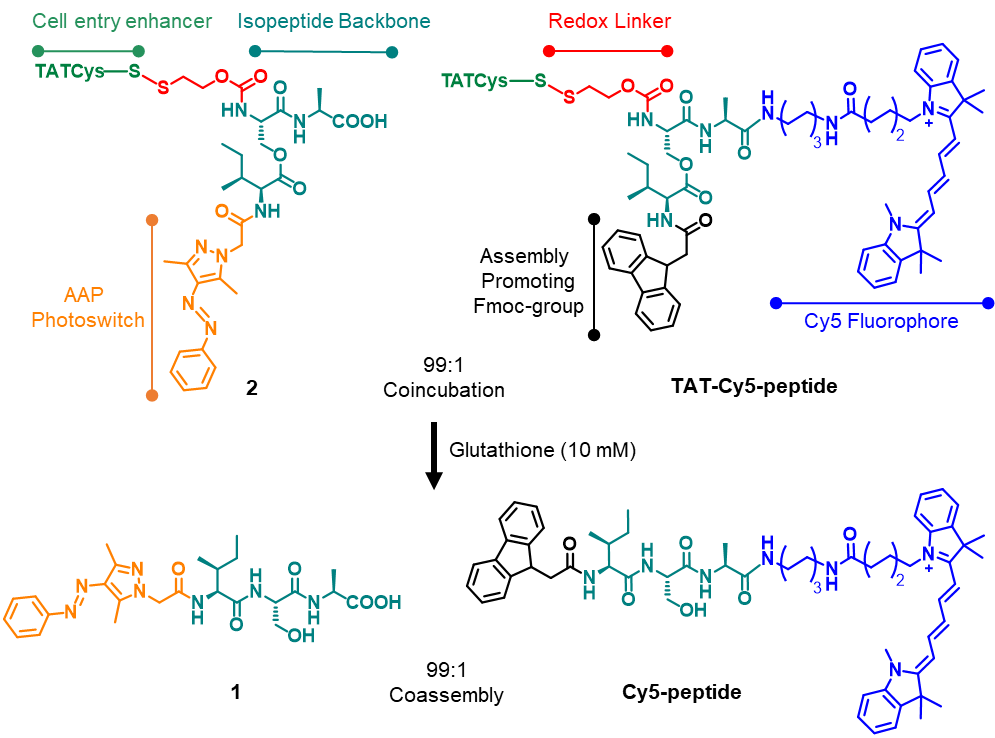


Figure S27: Chemical Design of the coassembly approach. Maintaining the isopeptide backbone, disulfide linker and cell-penetrating TAT-sequence, the AAP photoswitch was changed to an assembly promoting Fmoc-unit to circumvent energy transfer from Cy5, that was connected to the *C*-terminus of the peptide to enable fluorescence.^2^ Cells were treated with the 99/1 coincubation of **2** with **TAT-Cy5-peptide**. Intracellular glutathione induces linker cleavage and subsequent rearrangement into **1** and **Cy5-peptide**, that coassemble into fluorescent nanostructures.

Procedure:

DMSO stock solutions of **2** and **TAT-Cy5-peptide** were prepared at 40 mM concentration. Then the stocks were combined at a ratio of 99/1 (**2/TAT-Cy5-peptide**) maintaining an overall peptide concentration of 40 mM. This stock was further diluted with DMSO to 20 mM or 10 mM respectively. Sample solutions were further diluted to an end concentration of 400, 200 or 100 µM with DMEM and added to the cells (total DMSO content = 1.00 %).

## 2.6 Coassembly Analysis


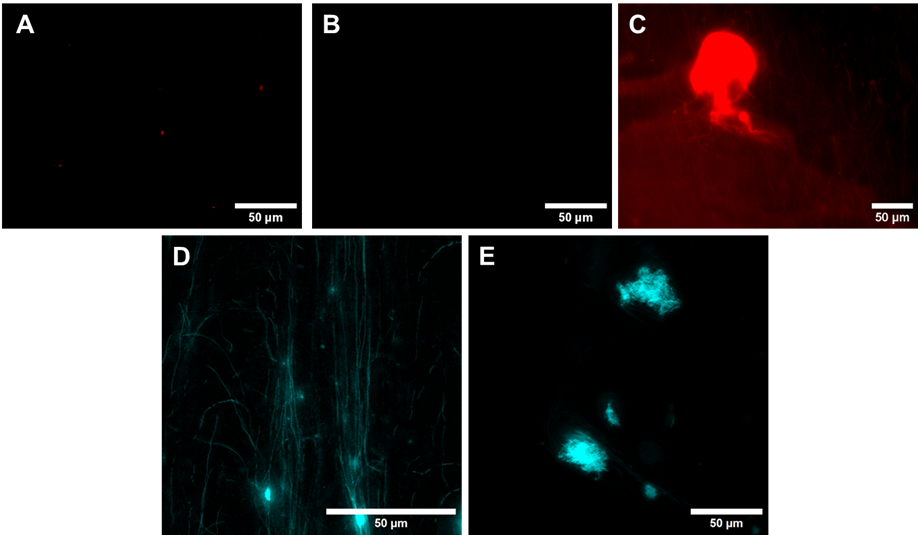


Figure S28: Wide-field fluorescence microscopy of the **Cy5-peptide** in the utilized effective concentration (2 µM) (**A**), of **1_trans_** (200 µM) (**B**) and of the coassembly of **1_trans_** with the **Cy5-peptide** (**C**, 99:1, 200 µM). Confocal laser scanning microscopy of of the coassembly of **1_trans_** with the **Cy5-peptide** (**D**, 99:1, 400 µM) and of **1_cis_** with the **Cy5-peptide** (**E**, 99:1, 400 µM). Magnification of the coassembly of **1_trans_** with the **Cy5-peptide** (**F**, 99:1, 200 µM).

Procedure:

DMSO stock solutions of **1*_trans_*** and **Cy5-peptide** were prepared at 40 or 20 mM concentration. For the analysis of **1*_cis_***, the DMSO stock was irradiated for 5 s with 365 nm light. Then the stocks were combined at a ratio of 99/1 (**1/Cy5-peptide**) maintaining an overall peptide concentration of 40 or 20 mM. Sample solutions were further diluted to an end concentration 400 or 200 µM with PBS (total DMSO content = 1 %) and imaged by placing a drop (5 µL) on a glass slide and covered with a cover slip. For the control experiments, **Cy5-peptide** (**A**, 2 µM) or **1** (**B**, 200 µM) were prepared without the addition of the respective other component in the effectively used concentration and imaged likewise.


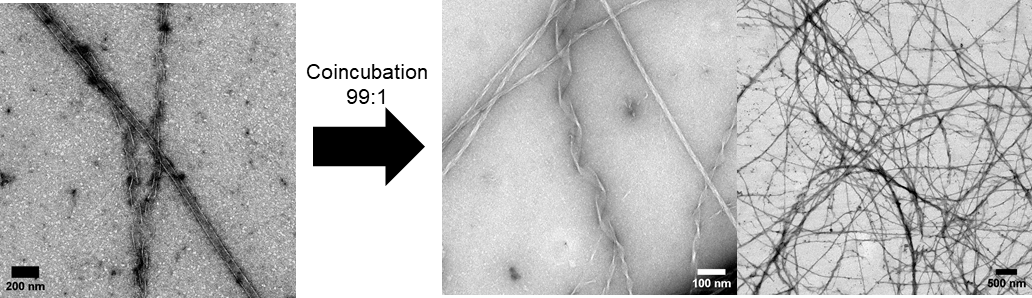


Figure S29: TEM micrographs of coassembly of **1*_trans_*** with the corresponding linearized **Cy5-peptide**. Left side shows data for only **1*_trans_***. Right side shows corresponding data for the 99/1 coincubation with **Cy5 peptide**. Overall peptide concentration is 200 µM.

## 2.7 ^1^H-NMR analysis of **1** with light switching


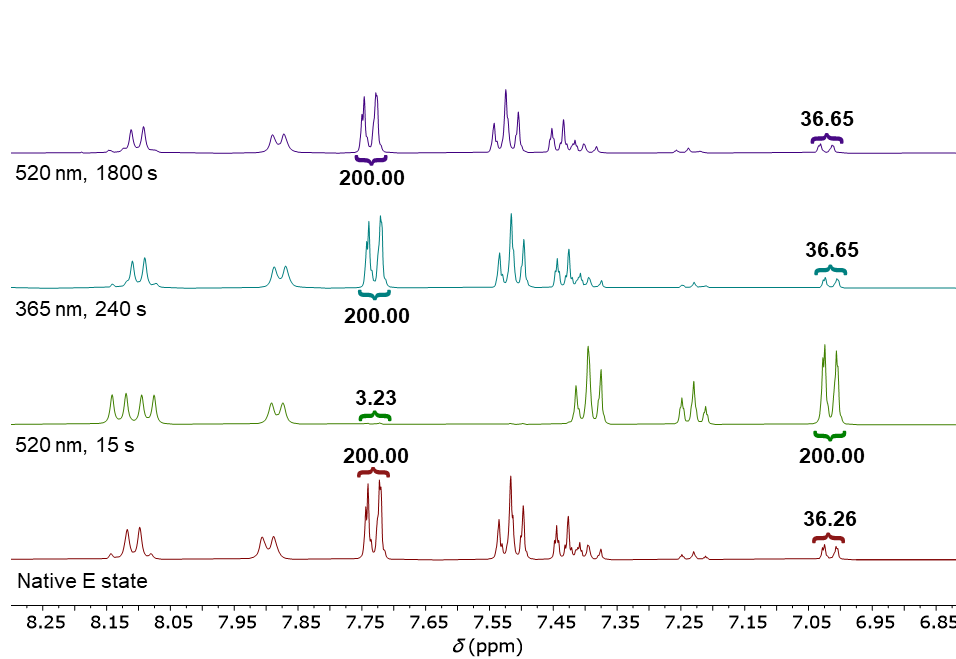


Figure S30: ^1^H-NMR spectroscopy (DMSO-d_6_, 298K, 400 MHz, 2 mg/ml) analysis of **1** in native state (red), after irradiation at 365 nm for 15 s (green), after subsequent irradiation at 520 nm for 240 s (blue) and subsequent irradiation at 520 nm for 1800 s. Integrals were set to 200.00 for the respective H3-protons of the predominant isomer.

## 2.8 LCMS Kinetic


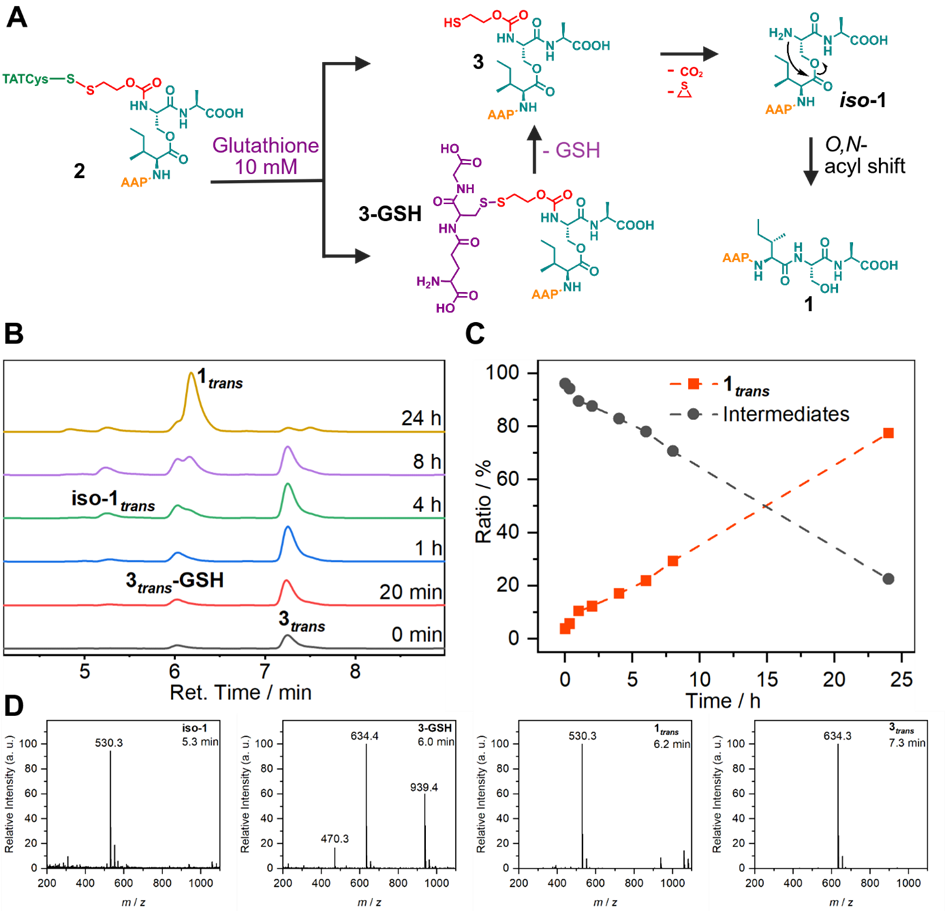


Figure S31: (**A**) Visualization of the chemical transformations within the bioresponsive reaction cascade. First, cleavage of the disulfide linker by glutathione, followed by self-immolation of the linker. Second, intramolecular rearrangement by *O,N*-acyl shift yielding the linearized AAP-ISA (**1**). (**B**) LC-MS kinetic analysis of the glutathione-induced linearization of **2_trans_** in NH_4_HCO_3_ buffer (50 mM, pH 7.4) and methanol (v/v 1:1) in the presence of intracellular concentrations of glutathione (10 mM) at room temperature. (**C**) Relative ratio of intermediates (**iso-1**, **3_cis_**, **3_trans_**, **3-GSH**) and final product **1_trans_** after the addition of glutathione-containing buffer, based on the peak integration at 300 nm. (**D**) Corresponding ESI MS spectra of the LCMS Kinetic.


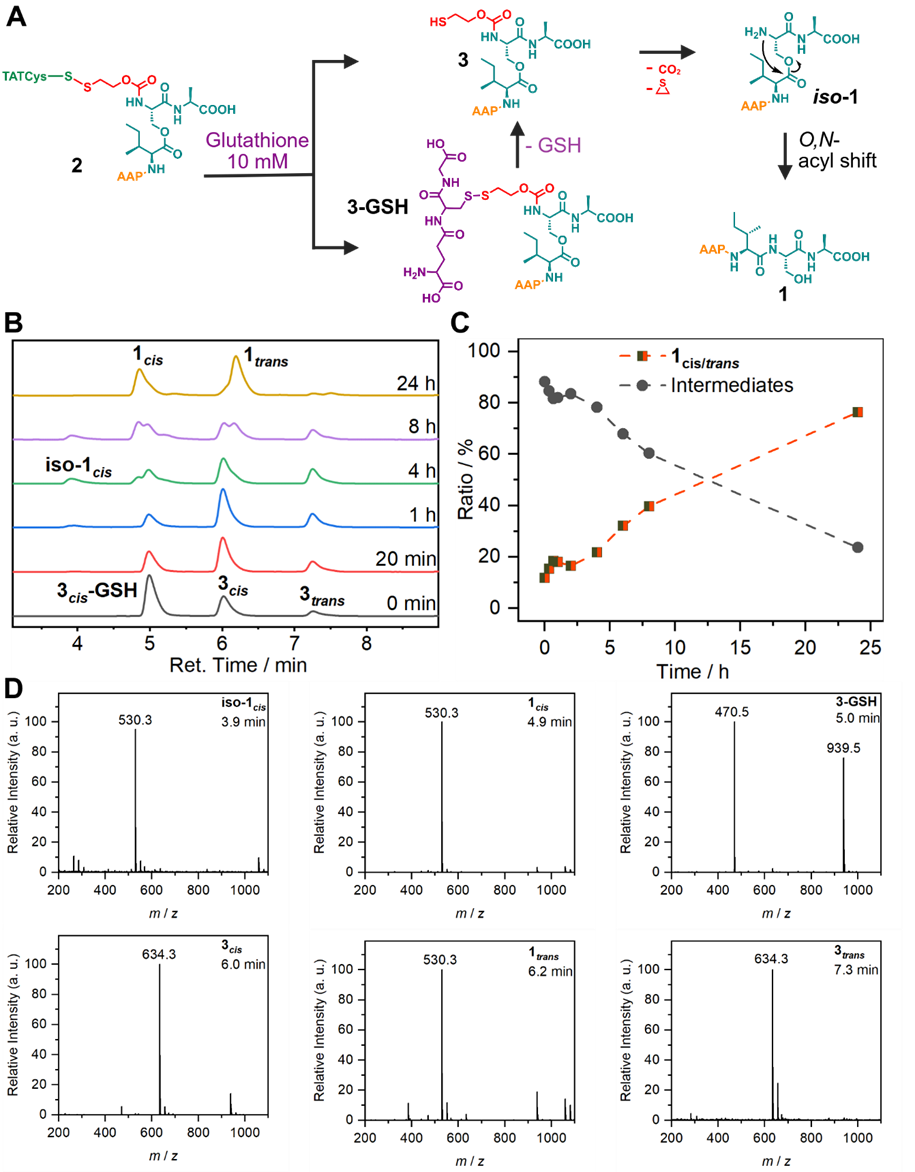


Figure S32: (**A**) Visualization of the chemical transformations within the bioresponsive reaction cascade. First, cleavage of the disulfide linker by glutathione, followed by self-immolation of the linker. Second, intramolecular rearrangement by *O,N*-acyl shift yielding the linearized AAP-ISA (**1**). (**B**) LC-MS kinetic analysis of the glutathione-induced linearization of **2_cis_** in NH_4_HCO_3_ buffer (50 mM, pH 7.4) and methanol (v/v 1:1) in the presence of intracellular concentrations of glutathione (10 mM) at room temperature. (**C**) Relative ratio of intermediates (**iso-1**, **3_cis_**, **3_trans_**, **3-GSH**) and final products **1_cis_** and **1_trans_** after the addition of glutathione-containing buffer, based on the peak integration at 300 nm. (**D**) Corresponding ESI MS spectra of the LCMS Kinetic.

## 2.9 Confocal Laser Scanning microscopy of 2 at various concentrations


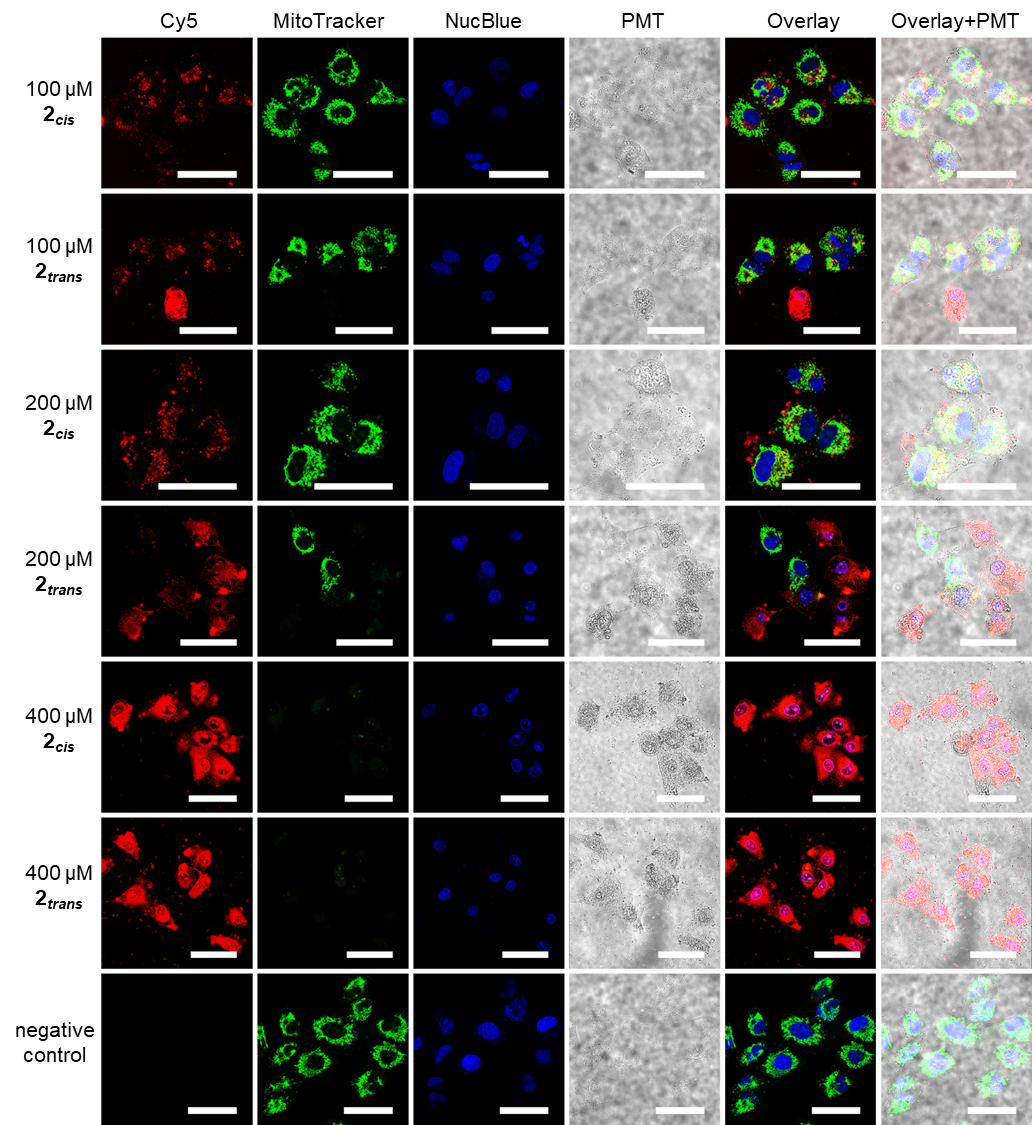


Figure S33: Confocal laser scanning micrographs of A549 cells treated for 4h with **2/Cy5-peptide** (99/1, 400, 200 or 100 µM) and prestained with Mitotracker Orange and NucBlue. Imaging under physiological condition (5% CO2, 90% humidty, 37°C) using Leica STELLARIS 8 microscope, HC PL APO CS2 40x/1.25 GLYC. Imaging: 550 nm at 2.13% for MitoTracker Orange excitation, 405 nm at 2.23% for NucBlue excitation and 650nm at 0.66% for sample excitation. Scale Bars: 50 µm.

# References

1 L. Stricker, E. C. Fritz, M. Peterlechner, N. L. Doltsinis and B. J. Ravoo, *J. Am. Chem. Soc.*, 2016, **138**, 4547–4554.

2 S. Chagri, K. Maxeiner, M. J. S. A. Silva, L. Förch, J. Link, P. Roth, R. Meyer, J. Fetzer, A. Kaltbeizel, I. Lieberwirth, K. Landfester, D. Y. W. Ng and T. Weil, *Adv. Sci.*, 2025, 2412606.
